# *p*-Terphenyl and Diphenyl Ether Derivatives from the Marine-Derived Fungus *Aspergillus candidus* HM5-4

**DOI:** 10.3390/md22010013

**Published:** 2023-12-24

**Authors:** Yanbo Zeng, Shirong Wang, Hanyang Peng, Weibo Zhao, Wenjun Chang, Hao Wang, Huiqin Chen, Haofu Dai

**Affiliations:** 1 Hainan Provincial Key Laboratory for Functional Components Research and Utilization of Marine Bio-Resources, Institute of Tropical Bioscience and Biotechnology, Chinese Academy of Tropical Agricultural Sciences & Key Laboratory for Biology and Genetic Resources of Tropical Crops of Hainan Province, Hainan Institute for Tropical Agricultural Resources, Haikou 571101, China; 13011987121@163.com (S.W.); 17379935106@163.com (H.P.); zwb1175642690@163.com (W.Z.); changwenjun@itbb.org.cn (W.C.); wanghao@itbb.org.cn (H.W.); chenhuiqin@itbb.org.cn (H.C.); 2 Ocean College of Hebei Agricultural University, Qinhuangdao 066000, China; 3 Zhanjiang Experimental Station of Chinese Academy of Tropical Agricultural Sciences, Zhanjiang 524013, China; 4 Jiangsu Key Laboratory for Functional Substances of Chinese Medicine, Nanjing University of Chinese Medicine, Nanjing 210023, China; 5 Beijing Key Laboratory for Separation and Analysis in Biomedicine and Pharmaceuticals, School of Life Science, Beijing Institute of Technology, Beijing 100081, China

**Keywords:** marine fungus, *Aspergillus candidus*, *p*-terphenyl, diphenyl ether, antifungal activity, cytotoxic activity, *α*-glucosidase inhibition

## Abstract

Two undescribed *p*-terphenyl derivatives, asperterphenylcins A–B (**1**–**2**), and two undescribed diphenyl ether derivatives, asperdiphenylcins A–B (**3**–**4**), together with three previously described *p*-terphenyl derivatives—4″-deoxyterprenin (**5**), terphenyllin (**6**), and 3″-hydroxyterphenyllin (**7**)—were obtained from the solid-rice culture of the marine-derived fungus *Aspergillus candidus* HM5-4, which was isolated from sponges from the South China Sea. Their structures were elucidated by HRESIMS data and NMR spectroscopic analysis. Compound **1** showed a strong inhibitory effect on *Neoscytalidium dimidiatum*, with an inhibition circle diameter of 31.67 ± 2.36 mm at a concentration of 10.0 µg/disc. Compounds **5** and **7** displayed cytotoxic activity against human chronic myeloid leukemia cells (K562), human liver cancer cells (BEL-7402), human gastric cancer cells (SGC-7901), human non-small cell lung cancer cells (A549) and human HeLa cervical cancer cells, with IC_50_ values ranging from 3.32 to 60.36 µM, respectively. Compounds **2**, **6** and **7** showed potent inhibitory activity against *α*-glucosidase, with IC_50_ values of 1.26 ± 0.19, 2.16 ± 0.44 and 13.22 ± 0.55 µM, respectively.

## 1. Introduction

Terphenyls are an important family of natural products, including *o*-terphenyls, *m*-terphenyls, and *p*-terphenyls [1]. Among these, *p*-terphenyls are the most frequently occuring natural terphenyls, and the most conspicuous feature of their structure is that two terminal benzene rings and a central ring make up their *para* arrangement [2,3]. Their structural diversity results from the different substituted functional groups on the three aromatic rings and the different connection modes between the rings [4]. Over 250 *p*-terphenyl derivatives have been identified, mainly from actinomyces, mosses and macrofungi [1]. In most cases, these secondary metabolites exhibit extensive biological activity, such as antimicrobial [5,6], cytotoxicity [7,8], free radical scavenging [9,10], phosphodiesterase inhibitory [11] and *α*-glucosidase inhibitory activities [12].

**Figure 1:**
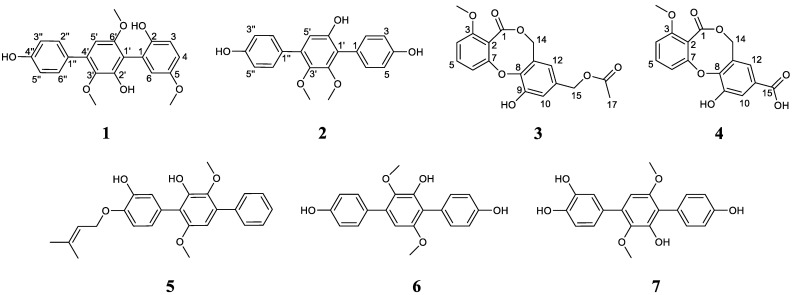
The structures of compounds **1**–**7,** obtained from *Aspergillus candidus* HM5-4: asperterphenylcins A–B (**1**,**2**), asperdiphenylcins A–B (**3**,**4**), 4″-deoxyterprenin (**5**), terphenyllin (**6**) and 3″-hydroxyterphenyllin (**7**).

The unique marine environment, with its high pressure, low temperature and high salinity, leads to the generation of secondary metabolites with different marine fungi biological activities, which are an important source of natural products with pharmacological effects [13]. Recently, a fair number of *p*-triphenyl derivatives with novel structures and good biological activity have been found from the *Aspergillus candidus* that is widely distributed in marine environments [7,14,15,16]. Almost all of these marine-derived *p*-triphenyls have cytotoxic effects, but some have neuroprotective and antibacterial effects. For this reason, in the process of searching for new bioactive natural products from marine-derived fungi [17,18,19,20], we carried out chemical studies on *A. candidus*, a fungus from a marine sponge which was collected from the coast of Lingao County, Hainan Province. Two undescribed *p*-terphenyl derivatives, asperterphenylcins A–B (**1**–**2**), and two undescribed diphenyl ether derivatives, asperdiphenylcins A–B (**3**–**4**), together with three previously described *p*-terphenyl derivatives—4″-deoxyterprenin (**5**), terphenyllin (**6**), and 3″-hydroxyterphenyllin (**7**; Figure 1)—were isolated from the crude EtOAc extract of a solid-rice culture of the marine sponge-derived fungus *A*. *candidus* HM5-4. Based on the extensive biological activity of *p*-terphenyls, all compounds were screened for antifungal, cytotoxic and *α*-glucosidase inhibitory activities. In this paper, we discuss the separation, structure identification and biological activities of these obtained compounds in detail.

## 2. Results

### 2.1. Structure Elucidation of New Compounds ***1***–***4***

**Figure 2:**
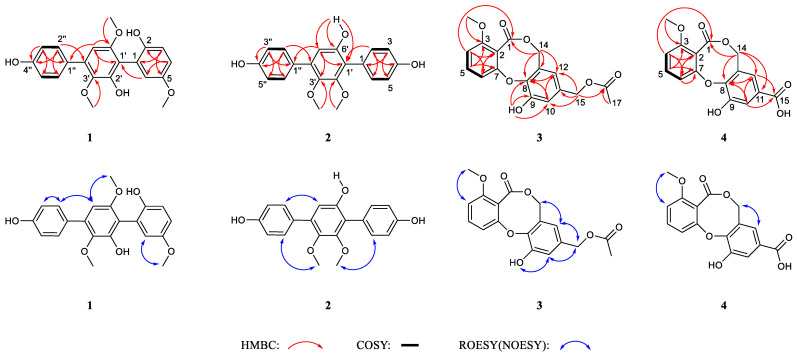
Key HMBC, ^1^H-^1^H COSY and ROESY(NOESY) correlations of compounds **1**–**4**.

Asperterphenylcin A (**1**) was isolated as a yellow film. Its molecular formula C_21_H_20_O_6_ was deduced by the HRESIMS ion peak at *m*/*z* 391.1139 (calcd. 391.1152 for C_21_H_20_O_6_Na, [M + Na]^+^; Figure S8), revealing twelve indexes of hydrogen deficiency. The characteristic IR spectrum absorption peak at 3415 cm^−1^ suggested the existence of a hydroxy group. The ^1^H NMR spectrum showed eight aromatic protons (from *δ*_H_ 6.38 to *δ*_H_ 7.43) and three methoxy groups (*δ*_H_ 3.29, *δ*_H_ 3.64 and *δ*_H_ 3.73) (Table 1). The ^13^C NMR spectrum revealed twenty-one carbon signals with the aid of the DEPT and HSQC spectra, which were attributed to eight protonated sp^2^ carbons (including two overlapping signals at (*δ*_C_ 115.1, *δ*_H_ 6.84) and (*δ*_C_ 129.7, *δ*_H_ 7.43), (*δ*_C_ 103.0, *δ*_H_ 6.38), (*δ*_C_ 114.7, *δ*_H_ 6.76), (*δ*_C_ 115.3, *δ*_H_ 6.83), (*δ*_C_ 123.5, *δ*_H_ 6.69)), ten non-protonated sp^2^ carbons (*δ*_C_ 117.1, *δ*_C_ 125.0, *δ*_C_ 128.7, *δ*_C_ 132.4, *δ*_C_ 139.3, *δ*_C_ 145.2, *δ*_C_ 146.7, *δ*_C_ 148.1, *δ*_C_ 153.1 and *δ*_C_ 156.7) and three methoxy carbons (*δ*_C_ 55.6, *δ*_C_ 55.7 and *δ*_C_ 60.0) (Table 1). Consideration of the co-isolated compounds **5**–**7** indicated that compound **1** was most likely a *p*-terphenyl derivative. The ^1^H-^1^H COSY correlations between *δ*_H_ 7.43 (2H, d, *J* = 8.0 Hz, H-2″/H-6″) and *δ*_H_ 6.84 (2H, d, *J* = 8.0 Hz, H-3″/H-5″) indicated the presence of a *p*-substituted benzene ring. The 1,2,5-trisubstituted benzene ring (benzene ring numbering) was established according to the ^1^H-^1^H COSY correlation between *δ*_H_ 6.69 (1H, d, *J* = 8.0 Hz, H-4) and *δ*_H_ 6.76 (1H, d, *J* = 8.0 Hz, H-3), with the aid of a key HMBC correlation between *δ*_H_ 6.83 (1H, s, H-6) and C-4 (*δ*_C_ 123.5), as shown in Figure 2. Finally, the remaining aromatic proton *δ*_H_ 6.38 (1H, s, H-5′) indicated the presence of a penta-substituted benzene ring. According to the molecular formula of **1**, the three methoxy groups contained three oxygen atoms, and the remaining oxygen atoms indicated the substitution of three phenolic hydroxyl groups. Ultimately, the positions of these substituent groups were determined, which were confirmed by the key HMBC correlations between H-5′ (*δ*_H_ 6.38) and C-1′ (*δ*_C_ 117.1), C-3′ (*δ*_C_ 139.3), C-6′ (*δ*_C_ 153.1) and C-1″ (*δ*_C_ 128.7), between H-2″ (*δ*_H_ 7.43) and C-4″ (*δ*_C_ 156.7) and C-4′ (*δ*_C_ 132.4), between H-3 (*δ*_H_ 6.76) and C-1 (*δ*_C_ 125.0) and C-5 (*δ*_C_ 146.7), and between H-4 (*δ*_H_ 6.69) and C-2 (*δ*_C_ 145.2) and C-6 (*δ*_C_ 115.3), along with the ROESY correlations between H-5′ (*δ*_H_ 6.38)/H-2″ (*δ*_H_ 7.43), H-5′ (*δ*_H_ 6.38)/H-6′-OCH_3_ (*δ*_H_ 3.64) and H-6 (*δ*_H_ 6.83)/H-5-OCH_3_ (*δ*_H_ 3.73). Thus, the structure of compound **1** was established as depicted in Figure 1.

Asperterphenylcin B (**2**) was isolated as a yellowish oil with a molecular formula C_20_H_18_O_5_, as determined by HRESIMS data, which displayed the ion peak at *m*/*z* 361.1039 (calcd. 361.1046 for C_20_H_18_O_5_Na, [M + Na]^+;^ Figure S18). A comparison of the ^1^H and ^13^C NMR spectra for compounds **2** and **1** implied they have similar structure, except that **2** has one less methoxy group than **1** (Table 1). The mass of **2** was 30 units lower than that of **1**, which further confirmed the above speculation. The ^1^H-^1^H COSY correlations between *δ*_H_ 7.13 (H-2/H-6)/*δ*_H_ 6.77 (H-3/H-5) and *δ*_H_ 7.31 (H-2″/H-6″)/*δ*_H_ 6.81 (H-3″/H-5″) indicated the presence of two *p*-substituted benzene rings. Thus, the remaining aromatic proton *δ*_H_ 6.56 (1H, s, H-5′) also revealed the presence of penta-substituted benzene rings. The key HMBC correlations between 6′-OH (*δ*_H_ 9.06, s) and C-1′ (*δ*_C_ 121.9), C-5′ (*δ*_C_ 111.2) and C-6′ (*δ*_C_ 150.8) indicated that there is a hydroxyl substitution rather than a methoxy group at the C-6′ (*δ*_C_ 150.8) position. Furthermore, the HMBC correlations between H-2/H-6 (*δ*_H_ 7.13) and C-1′ (*δ*_C_ 121.9) and C-4 (*δ*_C_ 156.0), and between H-2″/H-6″ (*δ*_H_ 7.31) and C-4′ (*δ*_C_ 133.7) and C-4″ (*δ*_C_ 156.6) indicated that there were two hydroxyl groups at the C-4 and C-4″ positions, respectively. Finally, the remaining two methoxy groups were fused at the C-2′ and C-3′ positions which was evidenced by the remaining key HMBC correlations between H-2′-OCH_3_ (*δ*_H_ 3.49) and C-2′ (*δ*_C_ 151.5) and between H-3′-OCH_3_ (*δ*_H_ 3.45) and C-3′ (*δ*_C_ 142.9), respectively (Figure 2).

Asperdiphenylcin A (**3**) was obtained as an orange solid. Its molecular formula was determined as C_18_H_16_O_7_, with eleven degrees of unsaturation based on the HRESIMS data at *m*/*z* 367.0790, (calcd. 367.0788 for C_18_H_16_O_7_Na, [M + Na]^+^; Figure S28). The IR spectrum indicated typical absorption bands for hydroxyl (3445 cm^−1^) and ester carbonyl (1737 cm^−1^) groups. The ^1^H NMR spectrum showed the presence of one methyl (*δ*_H_ 2.04), one methoxy (*δ*_H_ 3.85), two oxymethylenes (*δ*_H_ 5.06 and 4.92) and five aromatic protons (*δ*_H_ 6.63, 6.78, 6.95, 7.09 and 7.56) (Table 2). The ^13^C NMR spectrum classified by the DEPT and HSQC spectra showed the resonances of one methyl (*δ*_C_ 20.7), one methoxyl (*δ*_C_ 56.3), two oxygenated methylenes (*δ*_C_ 64.7 and 68.2), five protonated sp^2^ carbons (*δ*_C_ 109.7, 114.2, 117.4, 119.1 and 133.9), seven non-protonated sp^2^ carbons (*δ*_C_ 115.0, 127.7, 132.7, 143.3, 148.8, 152.0 and 156.7) and two ester-carbonyls (*δ*_C_ 166.2 and 170.1) (Table 2). A detailed analysis of the 1D and 2D NMR data revealed that **3** most resembled barceloneic lactone B [21], which was obtained from the marine fungal strain *Aspergillus* sp. They shared the same molecular skeleton and had a diphenyl ether skeleton with an eight-membered lactone ring. The aromatic protons at *δ*_H_ 7.09 (1H, d, 8.4, H-4), *δ*_H_ 7.56 (1H, t, 8.3, H-5) and *δ*_H_ 6.78 (1H, d, 8.1, H-6) and the further observed ^1^H-^1^H COSY correlation signals between H-4/H-5/H-6 indicated the presence of a 1,2,3-trisubstituted benzene ring in **3**. The existence of another 1,2,3,5-tetrasubstituted benzene ring was indicated by the remaining two aromatic protons at *δ*_H_ 6.95 (1H, s, H-10) and *δ*_H_ 6.63 (1H, s, H-12) and the key HMBC correlations between *δ*_H_ 6.95 (1H, s, H-10) and C-8 (*δ*_C_ 143.3) and C-12 (*δ*_C_ 119.1). Based on the downfield shifts of C-7 (*δ*_C_ 152.0) and C-8 (*δ*_C_ 143.3), an oxygen bridge between the C-7 and C-8 positions was deduced. According to the long-range HMBC correlation between *δ*_H_ 7.09 (1H, d, 8.4, H-4) and C-1 (*δ*_C_ 166.2) and the key HMBC correlations between *δ*_H_ 5.06 (2H, s, H-14) and C-1 (*δ*_C_ 166.2), C-8 (*δ*_C_ 143.3), C-13 (*δ*_C_ 127.7) and C-12 (*δ*_C_ 119.1), the presence of a lactone ring between C-2 and C-14 was determined. Furthermore, the remaining methyl (*δ*_C_ 20.7), oxygenated methylene (*δ*_C_ 64.7) and ester-carbonyl (*δ*_C_ 170.1) indicated the presence of a methyl acetate group, which was further confirmed at the C-11 position by key HMBC correlations between *δ*_H_ 4.92 (2H, s, H-15) and C-10 (*δ*_C_ 117.4), C-11 (*δ*_C_ 132.7), C-12 (*δ*_C_ 119.1) and C-16 (*δ*_C_ 170.1), along with the correlation between *δ*_H_ 2.04 (3H, s, H-17) and C-16 (*δ*_C_ 170.1). Ultimately, the planar structure of **3** was elucidated as shown in Figure 1, which was further determined using other key HMBC correlations, shown in Figure 2.

Asperdiphenylcin B (**4**) was obtained as a white crystal. Its molecular formula was determined to be C_16_H_12_O_7_ by the HRESIMS data at *m*/*z* 655.1028 (calcd. 655.1058 for C_32_H_24_O_14_Na, [2M + Na]^+^; Figure S38), indicating the presence of two carbon and two hydrogen atoms less than **3**, with eleven degrees of unsaturation. The IR spectrum of **4** also showed the typical absorption bands of hydroxyl (3422 cm^−1^) and ester carbonyl (1736 cm^−1^) groups. A comprehensive analysis of the ^1^H NMR and ^13^C NMR spectra of **4** revealed that the NMR data of **4** almost resembled those of **3**, indicating that they almost shared an identical structure skeleton (Table 2). Specifically, the characteristic peaks in the ^13^C NMR spectrum of five protonated sp^2^ carbons (*δ*_C_ 110.8, 115.2, 119.4, 122.8 and 134.5), seven non-protonated sp^2^ carbons (*δ*_C_ 116.6, 127.9, 128.9, 148.3, 149.8, 152.9 and 158.3), one oxygenated methylene (*δ*_C_ 69.0) and one ester-carbonyl (*δ*_C_ 166.4) in **4** indicated the same diphenyl ether skeleton with an eight-membered lactone ring as in **3**, which was further confirmed by the similar key HMBC correlations in Figure 2. The only difference between **4** and **3** was the side chain at C-11, in the light of HMBC correlations between H-10 (*δ*_H_ 7.65) and C-15 (*δ*_C_ 166.6) and between H-12 (*δ*_H_ 7.39) and C-15 (*δ*_C_ 166.6), suggesting that there was a carboxyl group attached to the C-11 in **4** instead of the acetoxymethyl group observed in **3**. Thus, the structure of **4** was assigned (Figure 1).

The known compounds 4″-deoxyterprenin (**5**) [22], terphenyllin (**6**) [23] and 3″-hydroxyterphenyllin (**7**) [14] were identified by a comparison of their MS and NMR data with data previously reported in the literature.

### 2.2. Bioassays of Compounds

#### 2.2.1. Cytotoxic Detection Activity

All the compounds mentioned above were tested for their cytotoxic activities against human myeloid leukemia cells (K562), human liver cancer cells (BEL-7402), human gastric cancer cells (SGC-7901), human non-small cell lung cancer cells (A549) and human HeLa cervical cancer cells. As shown in Table 3, 4″-deoxyterprenin (**5**) and 3″-hydroxyterphenyllin (**7**) exhibited certain inhibitory activities against these five types of cancer cells. Compared to the cytotoxic activity of **7**, the cytotoxic activity of **5** was much stronger. The IC_50_ values of **7** were 6.69 ± 0.12 µM against BEL-7402, 3.67 ± 0.17 µM against SGC-7901, and 3.32 ± 0.10 µM against A549, which was close to the IC_50_ values of positive control cisplatin (4.02 ± 0.06 µM against BEL-7402, 4.11 ± 0.02 µM against SGC-7901, and 1.93 ± 0.02 µM against A549; Table 3). Comparing the structure of compounds **5** and **7** revealed that **5**’s cytotoxic activity was greatly enhanced, probably because of the exist of the isopentene group in **5**.

#### 2.2.2. *α*-Glucosidase Inhibitory Activity

All the above-mentioned compounds were subjected to an *α*-glucosidase inhibitory activity test by a reported method [24], using genistein as a positive control. The results indicated that compounds **2** and **6** displayed more potent inhibitory activity (IC_50_ values were 1.26 ± 0.19 and 2.16 ± 0.44 µM) than genistein (IC_50_ value was 13.04 ± 2.56 µM; Table 4). The inhibitory activity of compound **7** was comparable to that of the positive drug genistein. Compound **1** had the weakest inhibitory activity, but it was still 6.7-times more active than acarbose. As shown in Table 4, all the diphenyl ether derivatives (**3** and **4**) exhibited no *α*-glucosidase inhibitory activity and all the *p*-terphenyl derivatives (**1**, **2**, **6** and **7**) except **5** exhibited *α*-glucosidase inhibitory activity. Comparing the structure of *p*-terphenyl derivatives **1**, **2**, **5**, **6** and **7** revealed that the *α*-glucosidase inhibitory activity of **5** completely disappeared, probably because the hydrogen in the hydroxyl group was substituted by an isopentene group in **5**.

#### 2.2.3. Antifungal Activity

The antifungal activities of three known compounds (**5**–**7**) were determined by the filter paper disc agar diffusion method. The results revealed that only compound **5** showed inhibitory activity against *N. dimidiatum*, which can cause a serious disease of stem canker in red-fleshed dragon fruit (*Hylocereus polyrhizus*); the diameter of its inhibition zone was 31.67 ± 2.36 mm. Carbendazim, as a positive control, displayed inhibitory activity against *N*. *dimidiatum*; the diameter of its inhibition zone was 55.00 ± 0.82 mm.

## 3. Materials and Methods

### 3.1. General Experimental Procedures

The NMR spectra were recorded on a Bruker AVIII-500 NMR spectrometer (Bruker Corporation, Karlsruhe, Germany) and Bruker DRX-600 spectrometer (Bruker Biospin AG, Fällanden, Germany). The chemical shifts of ^1^H NMR (500 MHz, 600 MHz) and ^13^C NMR (125 MHz, 150 MHz,) data were shown in *δ* (ppm) and referenced against the solvent signal (DMSO-*d*_6_, *δ*_H_ 2.50 and *δ*_C_ 39.52; acetone-*d*_6_, *δ*_H_ 2.05 and *δ*_C_ 29.84). HRESIMS data were measured on an API QSTAR Pulsar mass spectrometer (Bruker, Bremen, Germany). UV and IR data were tested on a UV-2550 spectrometer (Shimadzu, Kyoto, Japan) and Nicolet 380 Infrared Spectrometer (Thermo Electron Corporation, Madison, WI, USA), respectively. The semi-preparative HPLC was conducted on a Waters 1525 HPLC equipped with an XBridge C_18_ column (5 μm, 250.0 mm × 10.0 mm; Waters Corporation, Milford, MA, USA). Thin-layer chromatography (TLC) was conducted on pre-coated glass plates (silica gel GF_254_, Qingdao Marine Chemical Inc., Qingdao, China). Column chromatography (CC) was conducted on silica gel (45–75 µm; Qingdao Marine Chemical Inc., Qingdao, China).

### 3.2. Fungal Material and Fermentation

The marine fungus *A. candidus* HM5-4 was isolated from sponges collected from the coast of Lingao County, Hainan Province. The molecular sequence of the strain was cloned using the primers ITS1 (TCCGTAGGTGAACCTGCGG) and ITS4 (TCCTCCGCTTATTGATATGC), and the ITS sequence of the strain was determined. The sequences were submitted to the BLAST database in NCBI (sequence number: OP550289) and were found to be 100% homologous to *A. candidus* (MH398542.1). Therefore, the fungus was identified as *Aspergillus candidus*, together with the colony morphology of the fungus. The fungal strain was preserved in the South China Sea Marine Fungus Bank, Institute of Tropical Bioscience and Biotechnology, Chinese Academy of Tropical Agriculture Sciences.

For chemical investigations, the marine fungal strain *A. candidus* HM5-4 was incubated on PDA medium (consisting of 200.0 g/L potato, 20.0 g/L glucose, 20.0 g/L agar, and 1000.0 mL deionized water) at 28 °C after being obtained from the South China Sea Marine Fungus Bank (maintained at −80 °C in an ultra-low temperature freezer). After three days, three pieces of media with fungi were transferred aseptically to three Erlenmeyer flasks with PDB media (consisting of 200.0 g/L potato, 20.0 g/L glucose, and 1000.0 mL deionized water) and incubated on a rotary shaker (180 rpm) at 28 °C for 72 h. Then, 2.0 mL of seed solution was added to one hundred 1000 mL Erlenmeyer flasks with rice solid media (each flask contained 80.0 g rice and 120.0 mL artificial seawater) for fermentation, respectively. These Erlenmeyer flasks were cultivated under static conditions at 28 °C for 30 days.

### 3.3. Extraction and Isolation

The whole fermentation cultures of the marine fungal strain *A. candidus* HM5-4 were ultrasonically extracted with ethyl acetate (EtOAc) at room temperature three times. The filtrates were combined and evaporated in vacuo to yield the crude extract of ethyl acetate (120.0 g). Then, the crude extract was homogeneously dispersed in water and extracted three times with petroleum ether, EtOAc and *n*-butanol, respectively, and then the extracts were evaporated in vacuo to obtain petroleum ether extract (12.0 g), EtOAc extract (10.0 g) and *n*-butanol extract (2.0 g). Then, the crude extract of EtOAc (10.0 g) was chromatographed using silica gel column chromatography (CC) via the gradient elution of petroleum ether/EtOAc (1:0, 9:1, 8:2, 7:3, 6:4, 1:1, 3:7, and 0:1, *v*/*v*) and CH_2_Cl_2_/MeOH (4:1 and 1:1, *v*/*v*) to give 21 fractions (Fr.1–Fr.21). Fr.6 (250.6 mg) was fractionated by Sephadex LH-20 CC (eluted with 100% MeOH) to give compound **5** (3.4 mg). Fr.13 (1.3 g) was re-chromatographed on silica gel CC eluted with petroleum ether/EtOAc (5:2, *v*/*v*) to give 8 subfractions (Fr.13.1–Fr.13.8). Fr.13.6 (501.7 mg) was fractionated by Sephadex LH-20 CC eluted with 100% MeOH to afford ten subfractions (Fr.13.6.1–Fr.13.6.10). Fr.13.6.4 (108.8 mg) was subjected to silica gel CC eluted with CH_2_Cl_2_/MeOH (110:1, *v*/*v*) to give 5 subfractions (Fr.13.6.4.1–Fr.13.6.4.5). Fr.13.6.4.2 (9.2 mg) was further purified by semi-preparative reverse-phase HPLC eluted with 55% MeOH to obtain compound **1** (2.2 mg *t*_R_ = 9.3 min). Fr.13.6.4.5 (4.5 mg) was re-chromatographed on silica gel CC eluted with CH_2_Cl_2_/MeOH (85:1; *v*/*v*) to yield compound **2** (2.5 mg). Fr.13.8 (572.9 mg) was fractionated by Sephadex LH-20 CC eluted with 100% MeOH to afford six subfractions Fr.13.8.1–Fr.13.8.6. Subfraction Fr.13.8.2 (16.8 mg) was further re-chromatographed on silica gel CC eluted with CH_2_Cl_2_/MeOH (130:1; *v*/*v*) to obtain compound **3** (2.4 mg). Fr.15 (4.6 g) was chromatographed over silica gel CC via the gradient elution of petroleum ether/EtOAc (1:0, 20:1, 10:1, 3:1, 1:1, and 0:1, *v*/*v*) to give 13 subfractions (Fr.15.1–Fr.15.13). Fr.15.9 (135.0 mg) was re-chromatographed on silica gel CC eluted with petroleum ether/CH_2_Cl_2_/MeOH (25:10:3; *v*/*v*/*v*) to yield compound **6** (95.1 mg). Fr.15.10 (1.9 g) was fractionated by Sephadex LH-20 CC eluted with 100% MeOH to yield ten subfractions Fr.15.10.1–Fr.15.10.10. Fr.15.10.6 (276.1 mg) was further re-chromatographed on silica gel CC eluted with petroleum ether/EtOAc (12:5; *v*/*v*) to afford 3 fractions (Fr.15.10.6.1–Fr.15.10.6.3). Fr.15.10.6.2 (12.5 mg) was purified by semi-preparative reverse-phase HPLC eluted with 45% MeOH to yield compound **7** (5.6 mg *t*_R_ = 8.6 min). Fr.15.10.6.3 (15.1 mg) was re-chromatographed on silica gel CC eluted with CH_2_Cl_2_/MeOH (110:1; *v*/*v*) to yield compound **4** (2.1 mg).

Asperterphenylcin A (**1**): yellow film; UV (MeOH) *λ*_max_ (log *ε*): 225 (2.79), 247 (2.55), 276 (2.80) nm; IR(KBr) *ν*_max_: 3415, 2929, 1612, 1398, 1236, 1076, 817 cm^−1^; ^1^H and ^13^C NMR data see Table 1; HRESIMS *m*/*z* 391.1139 [M + Na]^+^ (calculated for C_21_H_20_O_6_Na, 391.1152).

Asperterphenylcin B (**2**): yellowish oil; UV (MeOH) *λ*_max_ (log *ε*): 227 (2.81), 247 (2.60), 268 (2.83) nm; IR(KBr) *ν*_max_: 3415, 2925, 1608, 1521, 1461, 1398, 1244, 1054, 835 cm^−1^; ^1^H and ^13^C NMR data see Table 1; HRESIMS *m*/*z* 361.1039 [M + Na]^+^ (calculated for C_20_H_18_O_5_Na, 361.1046).

Asperdiphenylcin A (**3**): orange solid; UV (MeOH) *λ*_max_ (log *ε*): 223 (2.77), 257 (2.24), 284 (2.55) nm; IR(KBr) *ν*_max_: 3445, 2937, 1737, 1602, 1459, 1280, 1243, 1079 cm^−1^; ^1^H and ^13^C NMR data see Table 2; HRESIMS *m*/*z* 367.0790 [M + Na]^+^ (calculated for C_18_H_16_O_7_Na, 367.0788).

Asperdiphenylcin B (**4**): white solid; UV (MeOH) *λ*_max_ (log *ε*): 222 (2.79), 241 (2.58), 246 (2.59), 268 (2.15), 291 (2.48) nm; IR(KBr) *ν*_max_: 3422, 1736, 1602, 1479, 1243, 1078, 763, 414cm^−1^; ^1^H and ^13^C NMR data see Table 2; HRESIMS *m*/*z* 655.1028 [2M + Na]^+^ (calculated for C_32_H_24_O_14_Na, 655.1058).

### 3.4. Cytotoxic Assay

All above-mentioned compounds were evaluated for their cytotoxicity in five human tumor cell lines: K562, BEL-7402, SGC-7901, A549 and HeLa cells which were bought from the Cell Bank of Type Culture Collection of the Shanghai Institute of Cell Biology, Chinese Academy of Sciences, using modified MTT methods [25]. The details of this are described in our previous paper [19]. The positive control was cisplatin.

### 3.5. α-Glucosidase Inhibition Assay

All abovementioned compounds were measured for their inhibitory effects against *α*-glucosidase, using PNPG as the substrate. The enzyme inhibitory assay was carried out by a formerly described method, with some modifications [24]. Compounds were dissolved in DMSO and six concentration gradients were set in turn. All the assays were performed in 0.1 M sodium phosphate buffer (PH = 6.8). The 10 μL sample was mixed with 100 μL *α*-glucosidase solution (0.2 U/mL, Sigma, Kanagawa, Japan) and shaken well, then added to a 96-well plate and incubated at 37 °C for 15 min. Subsequently, 40 μL of 2.5 mM 4-nitrophenyl-α-D-glucopyranoside was added and further incubated at 37 °C for 15 min. DMSO was used as a control and the blank wells contained buffer in place of substrate. The OD values were tested at 405 nm with a microplate reader. The positive control was genistein. The percentage inhibition was calculated by the following equation:

% inhibition = ((OD_control_ − OD_sample_)/(OD_control_ − OD_blank_)) × 100

### 3.6. Antifungal Activity Test

The inhibitory activities of some compounds against six phytopathogenic fungi (*Fusarium oxysporum* f. sp. cubense, *Colletotrichum gloeosporioides*, *Thielaviopsis paradoxa*, *Bipolaris cactivora* (Petrak) Alcorn, *Neoscytalidium dimidiatum*, and *Colletotrichum scovillei*) were evaluated using the filter paper disc agar diffusion method [26]; 250 μL of the fungal solution was aspirated onto the corresponding solid medium and spread evenly with a cotton swab. Samples were dissolved in MeOH to prepare solutions of 1.0 mg/mL and 3.0 mg/mL concentrations. Carbendazim (10 μL, 1.0 mg/mL) was used as a positive control and MeOH (10 μL) was used as negative control. The diameter of the inhibition zones of the plates cultured at 37 °C for 24 h were measured including the 6 mm disc diameter. Experiments were performed in triplicate. The results are showed as the mean value ± SD.

## 4. Conclusions

In summary, two undescribed *p*-terphenyl derivatives, asperterphenylcins A–B (**1**–**2**), and two undescribed diphenyl ether derivatives, asperdiphenylcins A–B (**3**–**4**), together with three previously described *p*-terphenyl derivatives—4″-deoxyterprenin (**5**), terphenyllin (**6**), and 3″-hydroxyterphenyllin (**7**)—were obtained from the crude EtOAc extract of a solid-rice culture of a marine sponge-derived fungus *A*. *candidus* HM5-4. Bioassays were used to evaluate its cytotoxic activity, *α*-glucosidase inhibitory activity and antifungal activity. The cytotoxic activity tests exhibited that 4″-deoxyterprenin (**5**) and 3″-hydroxyterphenyllin (**7**) showed certain inhibitory activities. Compared to the cytotoxic activity of **7**, the cytotoxic activity of **5** was much stronger. The IC_50_ values of **7** were 6.69 ± 0.12 µM against BEL-7402, 3.67 ± 0.17 µM against SGC-7901, and 3.32 ± 0.10 µM against A549, which was close to the IC_50_ values of the positive control cisplatin (4.02 ± 0.06 µM against BEL-7402, 4.11 ± 0.02µM against SGC-7901 and 1.93 ± 0.02 µM against A549). Comparing the structures of compounds **5** and **7** revealed that the cytotoxic activity of **5** was greatly enhanced, probably because of the exist of an isopentene group in **5**. The results of the antifungal assay showed that compound **5** has an obvious inhibitory effect on *N. dimidiatum*, which can cause a serious disease of stem canker in red-fleshed dragon fruit (*Hylocereus polyrhizus*); the diameter of its inhibition zone was 31.67 ± 2.36 mm. The bioassay results for the *α*-glucosidase inhibitory activity showed that compounds **2**, **6** and **7** exhibited potent *α*-glucosidase inhibitory activity, with IC_50_ values ranging from 1.26 ± 0.44 to 13.22 ± 0.55 μM. To the best of our knowledge, polyhydroxy phenolic compounds usually possess potent *α*-glucosidase inhibitory activity. Comparing the structure of *p*-terphenyl derivatives **1**, **2**, **5**, **6** and **7** revealed that the *α*-glucosidase inhibitory activity of **5** completely disappeared, probably because the hydrogen in the hydroxyl group was substituted by an isopentene group in **5**. In conclusion, *p*-terphenyl derivatives **2**, **6** and **7**, with potent *α*-glucosidase inhibitory activity, possess the potential to be developed as novel *α*-glucosidase inhibitors and need to be further studied.

## Figures and Tables

**Table 1:** ^1^H NMR (500 MHz) and ^13^C NMR (125 MHz) data of compounds **1**–**2**.

Position	1 ^a^		2 ^a^	
	*δ*_H_	*δ*_C_	*δ*_H_	*δ*_C_
1		125.0, C		124.6, C
2		145.2, C	7.13, d (7.9)	131.5, CH
3	6.76, d (8.0)	114.7, CH	6.77, d (7.9)	114.4, CH
4	6.69, d (8.0)	123.5, CH		156.0, C
5		146.7, C	6.77, d (7.9)	114.4, CH
6	6.83, s	115.3, CH	7.13, d (7.9)	131.5, CH
1′		117.1, C		121.9, C
2′		148.1, C		151.5, C
3′		139.3, C		142.9, C
4′		132.4, C		133.7, C
5′	6.38, s	103.0, CH	6.56, s	111.2, CH
6′		153.1, C		150.8, C
1″		128.7, C		128.5, C
2″	7.43, d (8.0)	129.7, CH	7.31, d (7.9)	129.7, CH
3″	6.84, d (8.0)	115.1, CH	6.81, d (3.9)	115.0, CH
4″		156.7, C		156.6, C
5″	6.84, d (8.0)	115.1, CH	6.81, d (3.9)	115.0, CH
6″	7.43, d (8.0)	129.7, CH	7.31, d (7.9)	129.7, CH
5-OCH_3_	3.73, s	55.7, CH_3_		
2′-OCH_3_			3.49, s	60.1, CH_3_
3′-OCH_3_	3.29, s	60.0, CH_3_	3.45, s	60.2, CH_3_
6′-OCH_3_	3.64, s	55.6, CH_3_		
4-OH			9.35, s	
6′-OH			9.06, s	
4″-OH			9.50, s	

^a^ Recorded in DMSO-*d*_6_.

**Table 2:** ^1^H NMR and ^13^C NMR data of compounds **3**–**4**.

Position	3 ^a^		4 ^b^	
	*δ*_H_	*δ*_C_	*δ*_H_	*δ*_C_
1		166.2, C		166.4, C
2		115.0, C		116.6, C
3		156.7, C		158.3, C
4	7.09, d (8.4)	109.7, CH	7.12, d (8.5)	110.8, CH
5	7.56, t (8.3)	133.9, CH	7.57, t (8.3)	134.5, CH
6	6.78, d (8.1)	114.2, CH	6.86, d (8.2)	115.2, CH
7		152.0, C		152.9, C
8		143.3, C		148.3, C
9		148.8, C		149.8, C
10	6.95, s	117.4, CH	7.65, d (1.8)	119.4, CH
11		132.7, C		128.9, C
12	6.63, s	119.1, CH	7.39, d (1.7)	122.8, CH
13		127.7, C		127.9, C
14	5.06, s	68.2, CH_2_	5.22, s	69.0, CH_2_
15	4.92, s	64.7, CH_2_		166.6, C
16		170.1, C		
17	2.04, s	20.7, CH_3_		
3-OCH_3_	3.85, s	56.3, CH_3_	3.92, s	56.8, CH_3_
9-OH	9.92, s			

^a 1^H NMR (125 MHz) and ^13^C NMR (500 MHz) was recorded in DMSO-*d*_6_; ^b 1^H NMR (150 MHz) and ^13^C NMR (600 MHz) was recorded in acetone-*d*_6_.

**Table 3:** Cytotoxic activities of compounds **1**–**7**.

Compounds	IC_50_ ± SD(µM) ^a^				
	K562	BEL-7402	SGC-7901	A549	HeLa
**1**	–	–	–	–	–
**2**	–	–	–	–	–
**3**	–	–	–	–	–
**4**	–	–	–	–	–
**5**	15.03 ± 0.14	6.69 ± 0.12	3.67 ± 0.17	3.32 ± 0.10	44.77 ± 0.13
**6**	–	–	–	–	–
**7**	33.98 ± 0.40	44.19 ± 0.71	33.44 ± 0.23	60.36 ± 0.51	53.65 ± 0.73
Cisplatin ^b^	3.08 ± 0.05	4.02 ± 0.06	4.11 ± 0.02	1.93 ± 0.02	11.29 ± 0.15

^a^ Values represent means ± SD based on three parallel experiments; ^b^ positive control—no activity at a concentration of 20 μg/mL.

**Table 4:** *α*-Glucosidase inhibitory activities of compounds **1**–**7**.

Compounds	IC_50_ ± SD (µM) ^a^
**1**	122.00 ± 3.30
**2**	1.26 ± 0.19
**3**	–
**4**	–
**5**	–
**6**	2.16 ± 0.44
**7**	13.22 ± 0.55
Genistein ^b^	13.04 ± 2.56

^a^ Values represent means ± SD based on three parallel experiments; ^b^ positive control—no activity at a concentration of 200 μM.

## Data Availability

The authors declare that all relevant data supporting the results of this study are available within the article and its Supplementary Materials file, or from the corresponding authors upon request.

## Supplementary Materials

The following supporting information can be downloaded at: https://www.mdpi.com/article/10.3390/md22010013/s1, Figures S1–S40: 1D, 2D NMR, MS, UV, and IR spectra of compounds **1**–**4**.

## References

[1] Xu K., Gao Y., Li Y.L., Xie F., Zhao Z.T., Lou H.X. (2018). Cytotoxic *p*-terphenyls from the endolichenic fungus *Floricola striata*. J. Nat. Prod..

[2] Chen W.H., Zhang J.W., Qi X., Zhao K., Pang X.Y., Lin X.P., Liao S.R., Yang B., Zhou X.F., Liu S.W. (2021). *p*-Terphenyls as anti-HSV-1/2 agents from a deep-sea-derived *Penicillium* sp.. J. Nat. Prod..

[3] Takahashi S., Suda Y., Nakamura T., Matsuoka K., Koshino H. (2017). Total synthesis of kehokorins A–E, cytotoxic *p*-terphenyls. J. Org. Chem..

[4] Li W., Li X.B., Lou H.X. (2018). Structural and biological diversity of natural *p*-terphenyls. J. Asian Nat. Prod. Res..

[5] Li W., Gao W., Zhang M., Li Y.L., Li L., Li X.B., Chang W.Q., Zhao Z.T., Lou H.X. (2016). *p*-terphenyl derivatives from the endolichenic fungus *Floricola striata*. J. Nat. Prod..

[6] Zhu J.J., Li Z.Y., Lu H.H., Liu S.Q., Ding W.J., Li J.Z., Xiong Y.H., Li C.Y. (2021). New diphenyl ethers from a fungus *Epicoccum sorghinum* L28 and their antifungal activity against phytopathogens. Bioorg. Chem..

[7] Lin Y.K., Xie C.L., Xing C.P., Wang B.Q., Tian X.X., Xia J.M., Jia L.Y., Pan Y.N., Yang X.W. (2021). Cytotoxic *p*-terphenyls from the deep-sea-derived *Aspergillus candidus*. Nat. Prod. Res..

[8] Wang D.Y., Wang Y., Ouyang Y.F., Zhu W.M. (2019). Cytotoxic *p*-terphenyls from a marine-derived *Nocardiopsis* species. J. Nat. Prod..

[9] Choi D.C., Ki D.W., Kim J.Y., Lee I.K., Yun B.S. (2023). *p*-Terphenyl glucosides from the culture broth of *Phlebiopsis castanea*. J. Antibiot..

[10] Sofian F.F., Kikuchi N., Koseki T., Kanno Y., Uesugi S., Shiono Y. (2022). Antioxidant *p*-terphenyl compound, isolated from edible mushroom, *Boletopsis leucomelas*. Biosci. Biotechnol. Biochem..

[11] Guo Z.K., Abulaizi A., Huang L., Xiong Z.J., Zhang S.Q., Liu T.M., Wang R. (2022). Discovery of *p*-terphenyl metabolites as potential phosphodiesterase PDE4D inhibitors from the coral-associated fungus *Aspergillus* sp. ITBBc1. Mar. Drugs.

[12] Xu Y.C., Wang Y., Wu D., He W.W., Wang L.P., Zhu W.M. (2021). *p*-terphenyls from *Aspergillus* sp. GZWMJZ-055: Identification, derivation, antioxidant and *α*-glycosidase inhibitory activities. Front. Microbiol..

[13] Shin H.J. (2020). Natural products from marine fungi. Mar. Drugs..

[14] Yurchenko E.A., Menchinskaya E.S., Pislyagin E.A., Chingizova E.A., Girich E.V., Yurchenko A.N., Aminin D.L., Mikhailov V.V. (2021). Cytoprotective activity of *p*-Terphenyl polyketides and flavuside B from marine-derived fungi against oxidative stress in Neuro-2a Cells. Molecules.

[15] Peng G.Y., Kurtán T., Mándi A., He J., Cao Z.Y., Tang H., Mao S.C., Zhang W. (2021). Neuronal modulators from the coral-associated fungi *Aspergillus candidus*. Mar. Drugs.

[16] Zhou G.L., Zhang X.M., Shah M., Che Q., Zhang G.J., Gu Q.Q., Zhu T.J., Li D.H. (2021). Polyhydroxy *p*-terphenyls from a mangrove endophytic fungus *Aspergillus candidus* LDJ-5. Mar. Drugs.

[17] Wang S., Zeng Y.B., Yin J.J., Chang W.J., Zhao X.L., Mao Y. (2022). Two new azaphilones from the marine-derived fungus *Penicillium sclerotiorum* E23Y−1A. Phytochem. Lett..

[18] Wang Z., Zeng Y.B., Zhao W.B., Dai F.H., Chang W.J., Lv F. (2022). Structures and biological activities of brominated azaphilones produced by *Penicillium sclerotiorum* E23Y−1A. Phytochem. Lett..

[19] Zeng Y.B., Wang Z., Chang W.J., Zhao W.B., Wang H., Chen H.Q., Dai F.H., Lv F. (2023). New azaphilones from the marine-derived fungus *Penicillium sclerotiorum* E23Y−1A with their anti-inflammatory and antitumor activities. Mar. Drugs.

[20] Zhao W.B., Zeng Y.B., Chang W.J., Chen H.Q., Wang H., Dai F.H., Lv F. (2023). Potential *α*-glucosidase inhibitors from the deep-sea sediment-derived fungus *Aspergillus insulicola*. Mar. Drugs.

[21] Liu S.S., Lu C.H., Huang J.J., Shen Y.M. (2012). Three new compounds from the marine fungal strain *Aspergillus sp*. AF119. Rec. Nat. Prod..

[22] Wei H., Inada H., Hayashi A., Higashimoto K., Pruksakorn P., Kamada S., Arai M., Ishida S., Kobayashi P. (2007). Prenylterphenyllin and its dehydroxyl analogs, new cytotoxic substances from a marine-derived fungus *Aspergillus candidus* IF10. J. Antibiot..

[23] Shan T.J., Wang Y.Y., Wang S., Xie Y.Y., Cui Z.H., Wu C.Y., Sun J., Wang J., Mao Z.L. (2020). A new *p*-terphenyl derivative from the insect-derived fungus *Aspergillus candidus* Bdf-2 and the synergistic effects of terphenyllin. PeerJ.

[24] Yang L., Yang Y.L., Dong W.H., Li W., Wang P., Cao X., Yuan J.Z., Chen H.Q., Mei W.L., Dai H.F. (2019). Sesquiterpenoids and 2-(2-phenylethyl) chromones respectively acting as *α*-glucosidase and tyrosinase inhibitors from agarwood of an *Aquilaria* plant. J. Enzym. Inhib. Med. Chem..

[25] Mosmann T. (1983). Rapid colorimetic assay for cellular growth and survival: Application to proliferation and cytotoxicity assays. J. Immunol. Methods.

[26] Li W.H., Ding L.J., Li J., Wen H.M., Liu Y., Tan S.L., Yan X.J., Shi Y.T., Lin W.H., He S. (2022). Novel antimycin analogues with agricultural antifungal activities from the sponge-associated actinomycete *Streptomyces* sp. NBU3104. J. Agr. Food. Chem..

