# Supplementary material for: p-Terphenyl and Diphenyl Ether Derivatives from the Marine-Derived Fungus Aspergillus candidus HM5-4
Source: Mar Drugs. 2023 Dec 24;22(1):13. doi: 10.3390/md22010013 (PMC10817493; doi:10.3390/md22010013)
Supplement: Supplementary file 1 [file marinedrugs-22-00013-s001.zip › marinedrugs-2755257-supplementary.pdf]

Supporting Information

p-Terphenyl and Diphenyl Ether Derivatives from Marine-Derived Fungus *Aspergillus candidus* HM5-4

Yanbo Zeng [1,2,5,†,*], Shirong Wang [1,2,†], Hanyang Peng [1,3], Weibo Zhao [1,4], Wenjun Chang [1,5], Hao Wang [1], Huiqin Chen[1], and Haofu Dai[1,*]

[1] *Hainan Provincial Key Laboratory for Functional Components Research and Utilization of Marine Bio-resources, Institute of Tropical Bioscience and Biotechnology, Chinese Academy of Tropical Agricultural Sciences & Key Laboratory for Biology and Genetic Resources of Tropical Crops of Hainan Province, Hainan Institute for Tropical Agricultural Resources, Haikou 571101, China; 13011987121@163.com (S.W.); 17379935106@163.com (H.P.); zwb1175642690@163.com (W.Z.); changwenjun@itbb.org.cn (W.C.); wanghao@itbb.org.cn (H.W.); chenhuiqin@itbb.org.cn (H.C.)*

[2] *Ocean College of Hebei Agricultural University, Qinhuangdao 066000, China*

[3] *Jiangsu Key Laboratory for Functional Substances of Chinese Medicine, Nanjing University of Chinese Medicine, Nanjing 210023, China*

[4] *Beijing Key Laboratory for Separation and Analysis in Biomedicine and Pharmaceuticals, School of Life Science, Beijing Institute of Technology, Beijing, 100081, China*

[5] *Zhanjiang Experimental Station of Chinese Academy of Tropical Agricultural Sciences, Zhanjiang, 524013, China*

[*] Correspondence: zengyanbo@itbb.org.cn (Y.Z.); daihaofu@itbb.org.cn (H.D.)

[†] These authors contributed equally to this work.

Figure S1. ^{1}H NMR (500 MHz, DMSO-d_6) spectrum of compound **1**

Figure S2. ^{13}C NMR (125 MHz, DMSO-d_6) spectrum of compound **1**

Figure S3. DEPT135 spectrum of compound **1**

Figure S4. HSQC spectrum of compound **1**

Figure S5. HMBC spectrum of compound **1**

Figure S6. ^{1}H-^{1}H COSY spectrum of compound **1**

Figure S7. ROESY spectrum of compound **1**

Figure S8. The HRESIMS spectrum of compound **1**

Figure S9. UV spectrum of compound **1**

Figure S10. IR spectrum of compound **1**

Figure S11. ^{1}H NMR (500 MHz, DMSO-d_6) spectrum of compound **2**

Figure S12. ^{13}C NMR (125 MHz, DMSO-d_6) spectrum of compound **2**

Figure S13. DEPT135 spectrum of compound **2**

Figure S14. HSQC spectrum of compound **2**

Figure S15. HMBC spectrum of compound **2**

Figure S16. ^{1}H-^{1}H COSY spectrum of compound **2**

Figure S17. ROESY spectrum of compound **2**

Figure S18. The HRESIMS spectrum of compound **2**

Figure S19. UV spectrum of compound **2**

Figure S20. IR spectrum of compound **2**

Figure S21. ^{1}H NMR (500 MHz, DMSO-d_6) spectrum of compound **3**

Figure S22. ^{13}C NMR (125 MHz, DMSO-d_6) spectrum of compound **3**

Figure S23. DEPT135 spectrum of compound **3**

Figure S24. HSQC spectrum of compound **3**

Figure S25. HMBC spectrum of compound **3**

Figure S26. ^{1}H-^{1}H COSY spectrum of compound **3**

Figure S27. ROESY spectrum of compound **3**

Figure S28. The HRESIMS spectrum of compound **3**

Figure S29. UV spectrum of compound **3**

Figure S30. IR spectrum of compound **3**

Figure S31. ^{1}H NMR (600 MHz, acetone-d_6) spectrum of compound **4**

Figure S32. ^{13}C NMR (150 MHz, acetone-d_6) spectrum of compound **4**

Figure S33. DEPT135 spectrum of compound **4**

Figure S34. HSQC spectrum of compound **4**

Figure S35. HMBC spectrum of compound **4**

Figure S36. ^{1}H-^{1}H COSY spectrum of compound **4**

Figure S37. ROESY spectrum of compound **4**

Figure S38. The HRESIMS spectrum of compound **4**

Figure S39. UV spectrum of compound **4**

Figure S40. IR spectrum of compound **4**

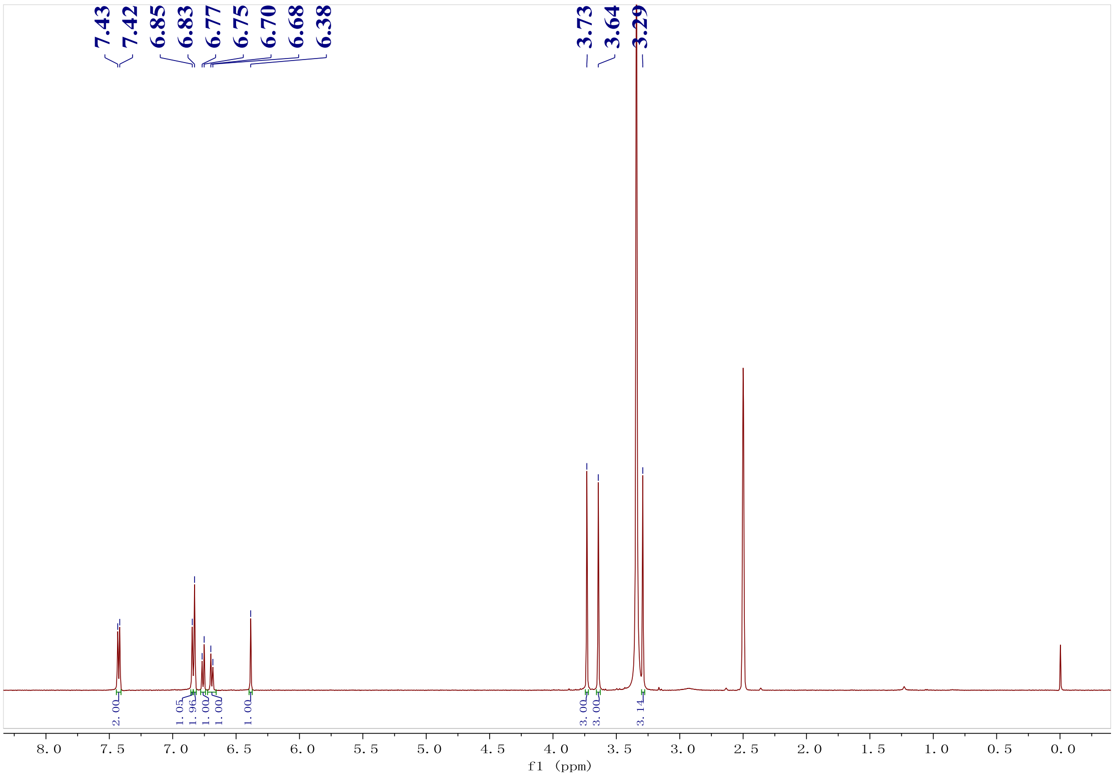

Figure S1. ^{1}H NMR (500 MHz, DMSO-d_6) spectrum of compound **1**

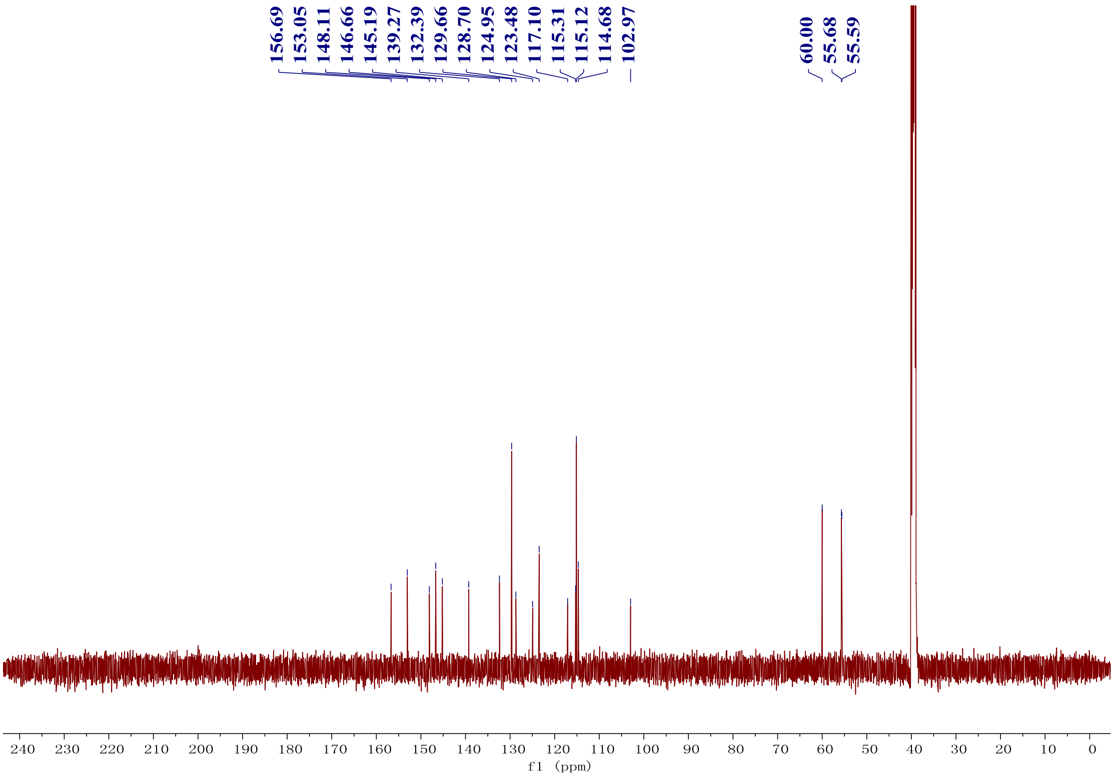

Figure S2. ^{13}C NMR (125 MHz, DMSO-d_6) spectrum of compound **1**

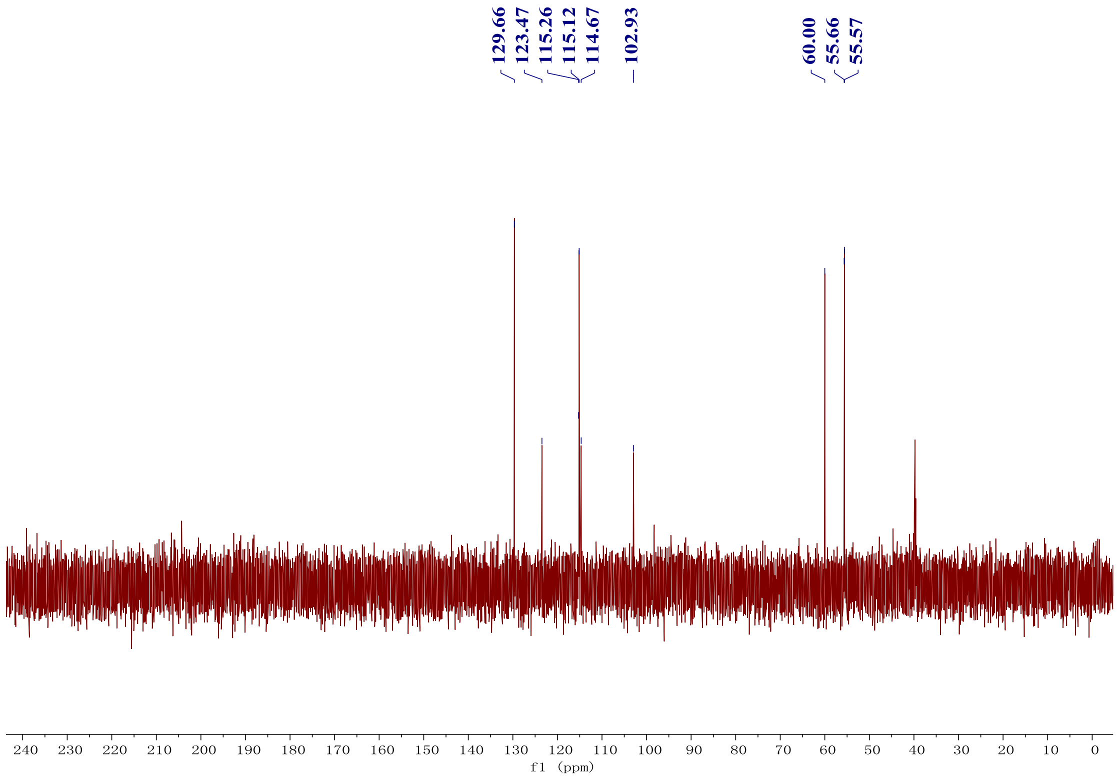

Figure S3. DEPT135 spectrum of compound **1**

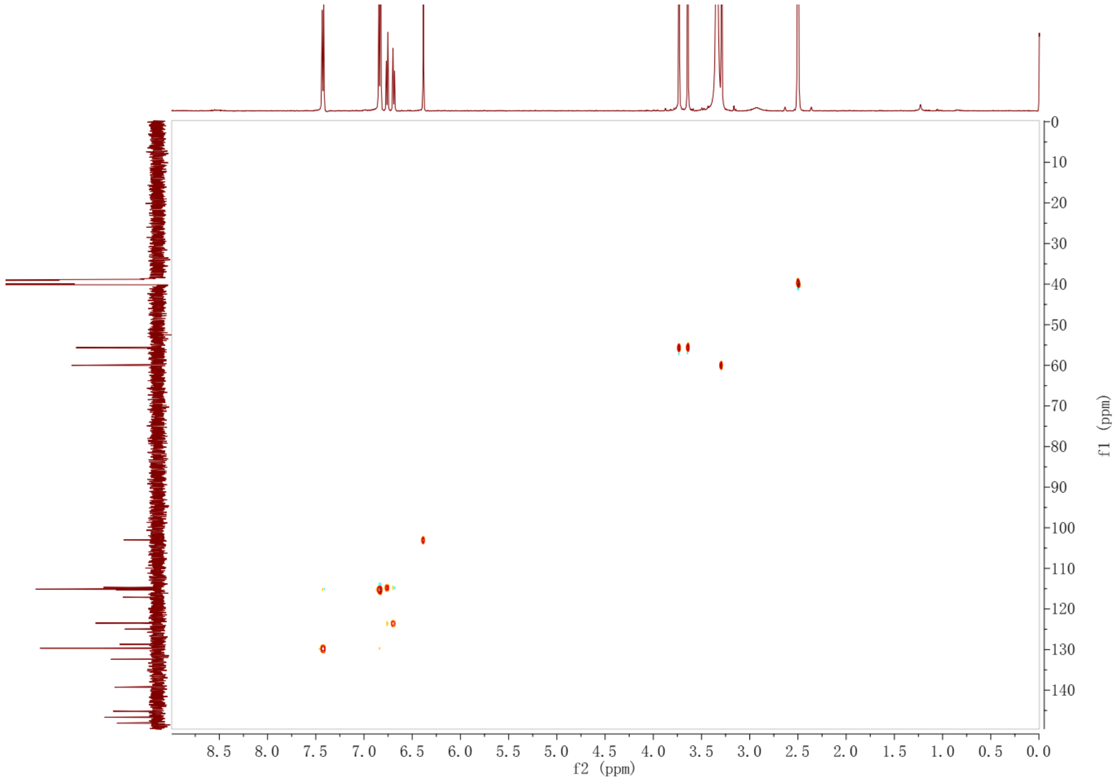

Figure S4. HSQC spectrum of compound **1**

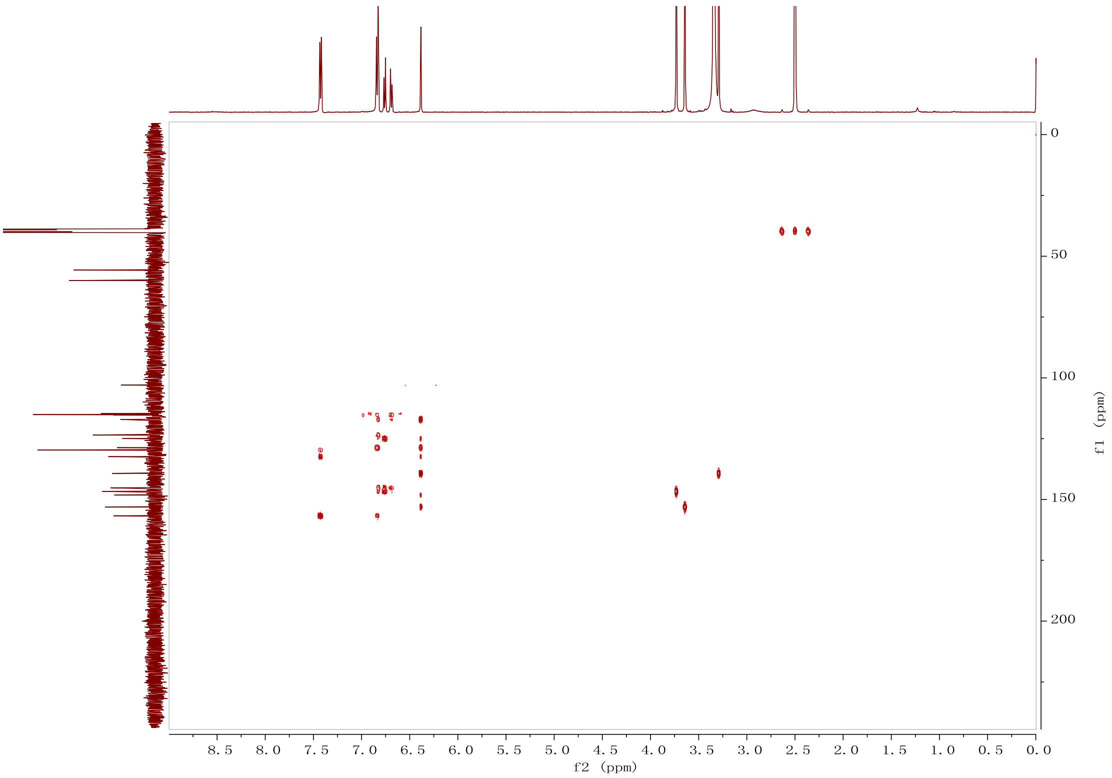

Figure S5. HMBC spectrum of compound **1**

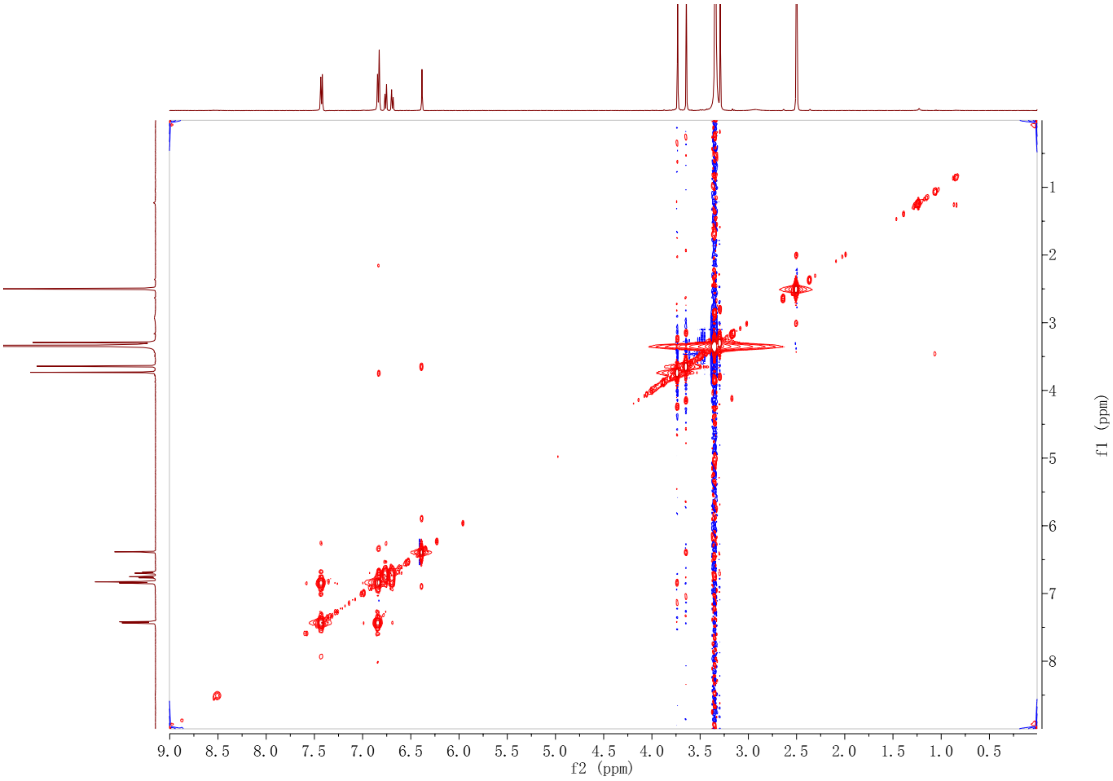

Figure S6. ^{1}H-^{1}H COSY spectrum of compound **1**

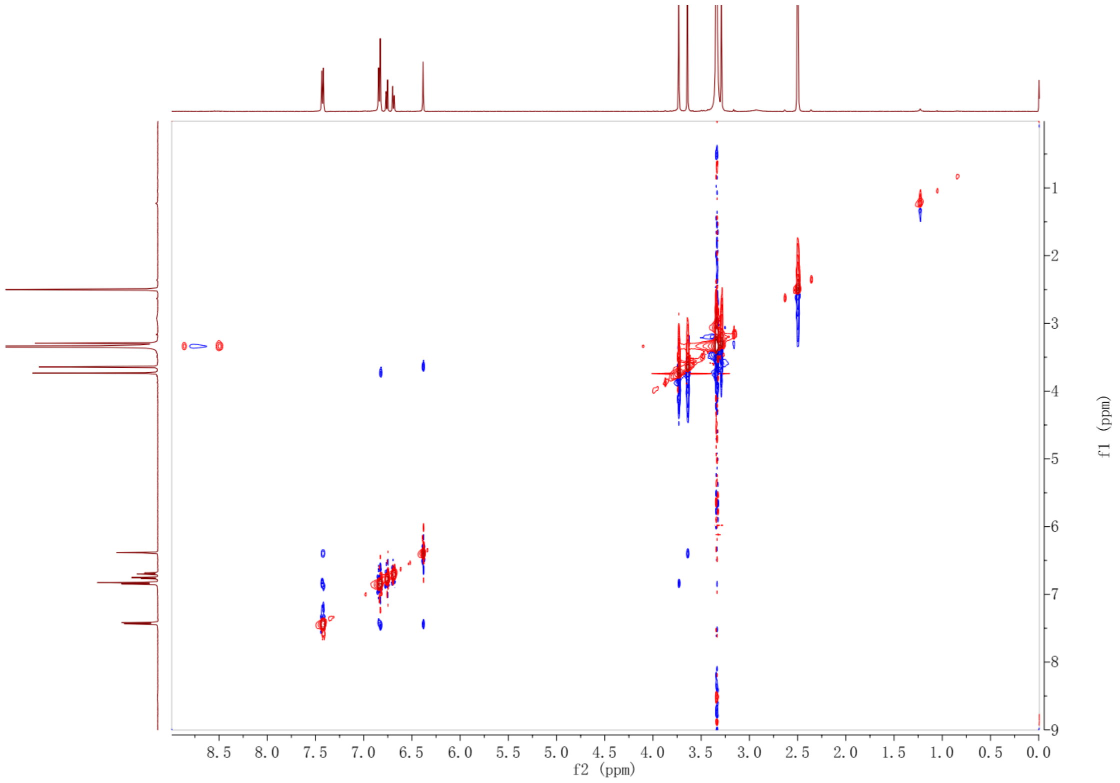

Figure S7. ROESY spectrum of compound **1**

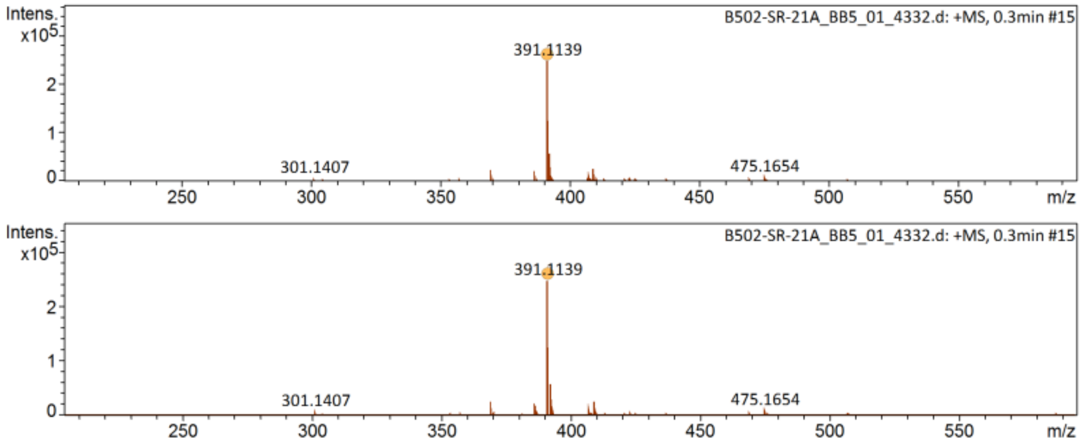

Meas. m/z	#	Ion Formula	m/z	err [ppm]	mSigma	# mSigma	Score	rdb	e^- Conf	N-Rule	Adduct
391.1139	1	C21H20NaO6	391.1152	3.4	2.7	1	100.00	12.0	even	ok	M+Na

Figure S8. The HRESIMS spectrum of compound **1**

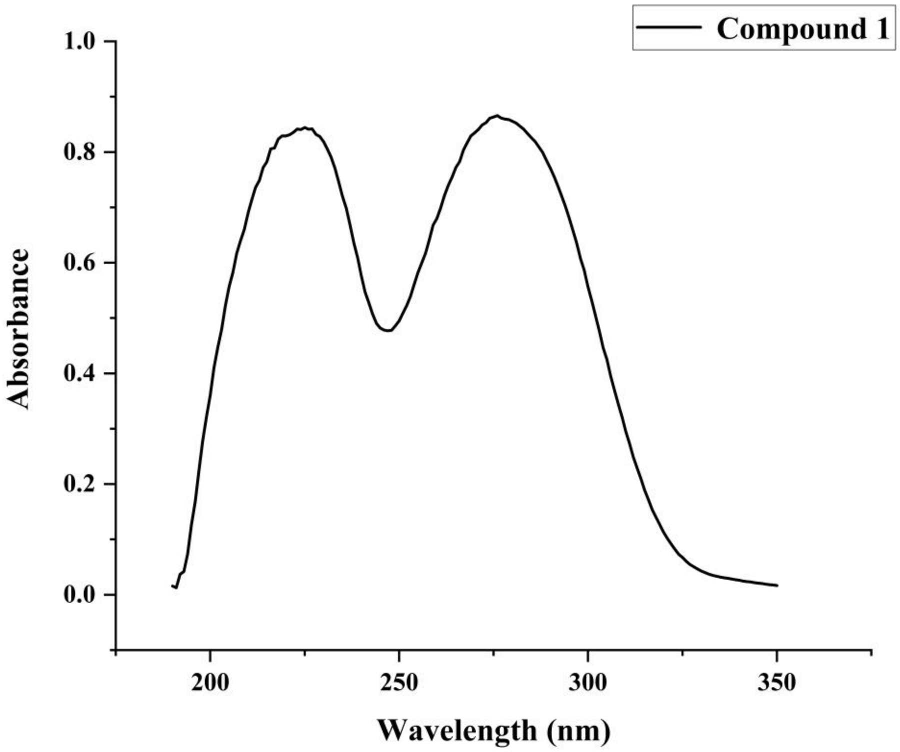

Figure S9. UV spectrum of compound **1**

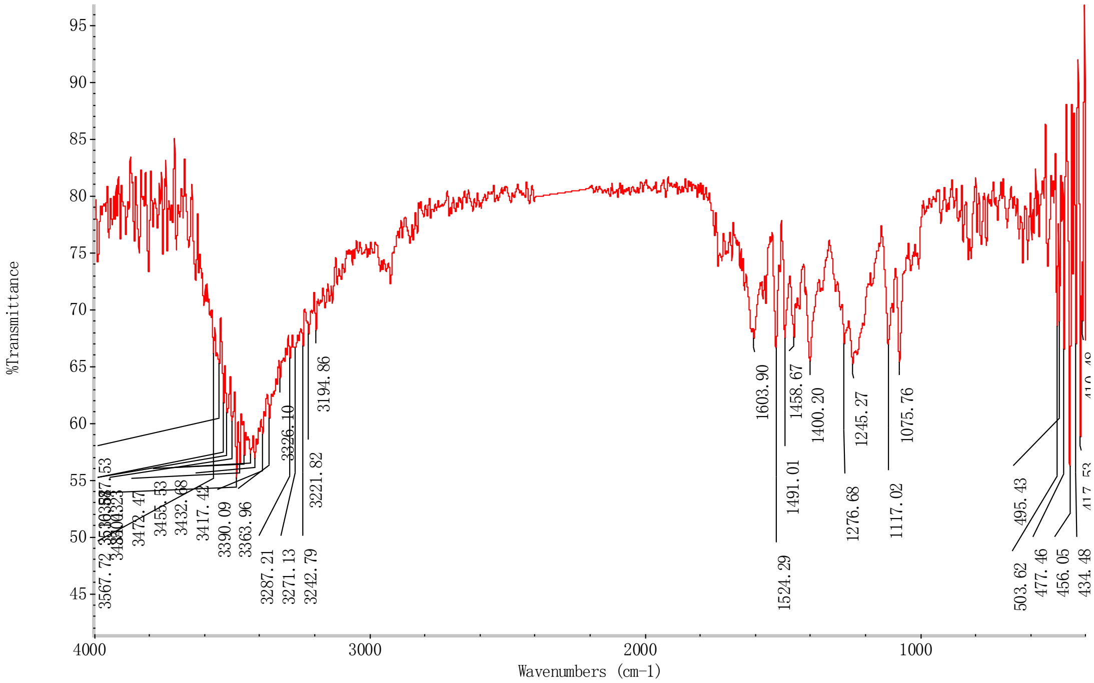

Figure S10. IR spectrum of compound **1**

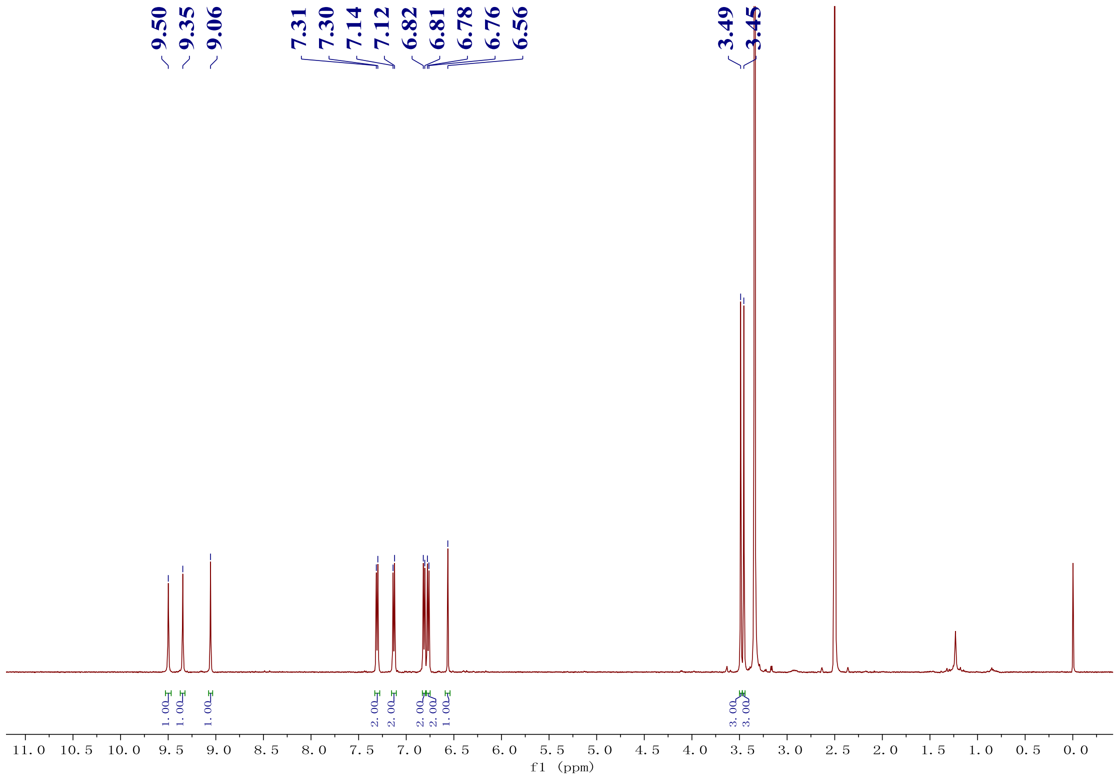

Figure S11. ^{1}H NMR (500 MHz, DMSO-d_6) spectrum of compound **2**

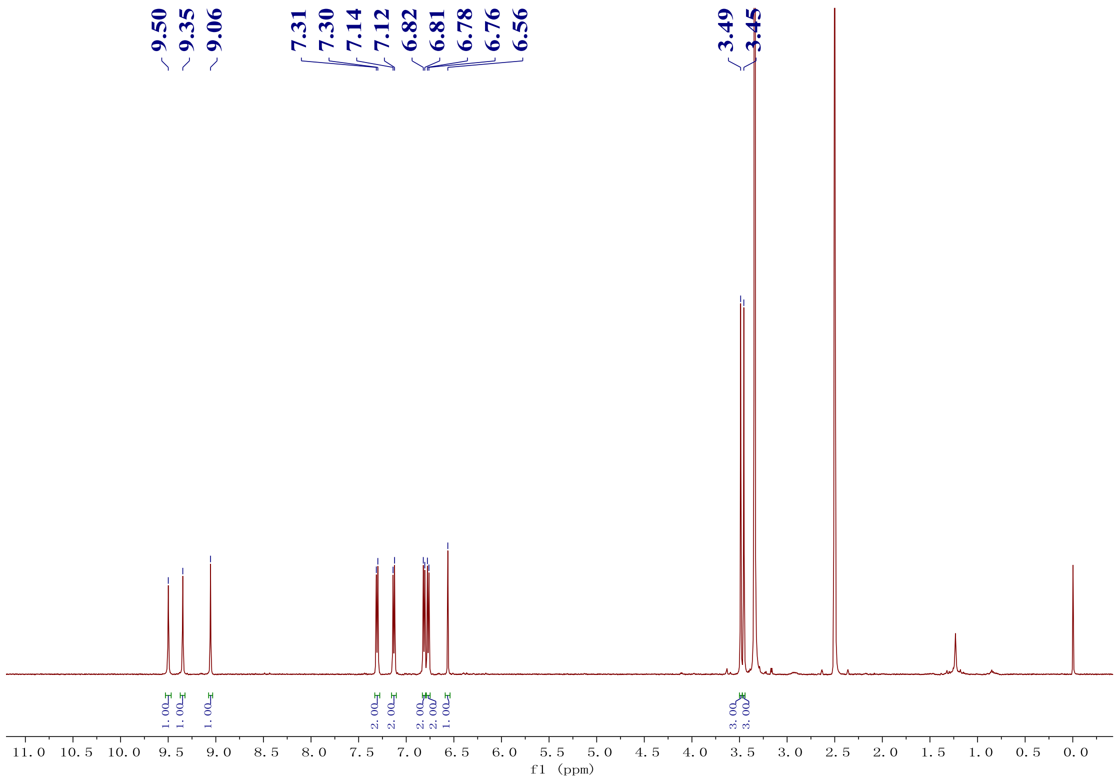

Figure S12. ^{13}C NMR (125 MHz, DMSO-d_6) spectrum of compound **2**

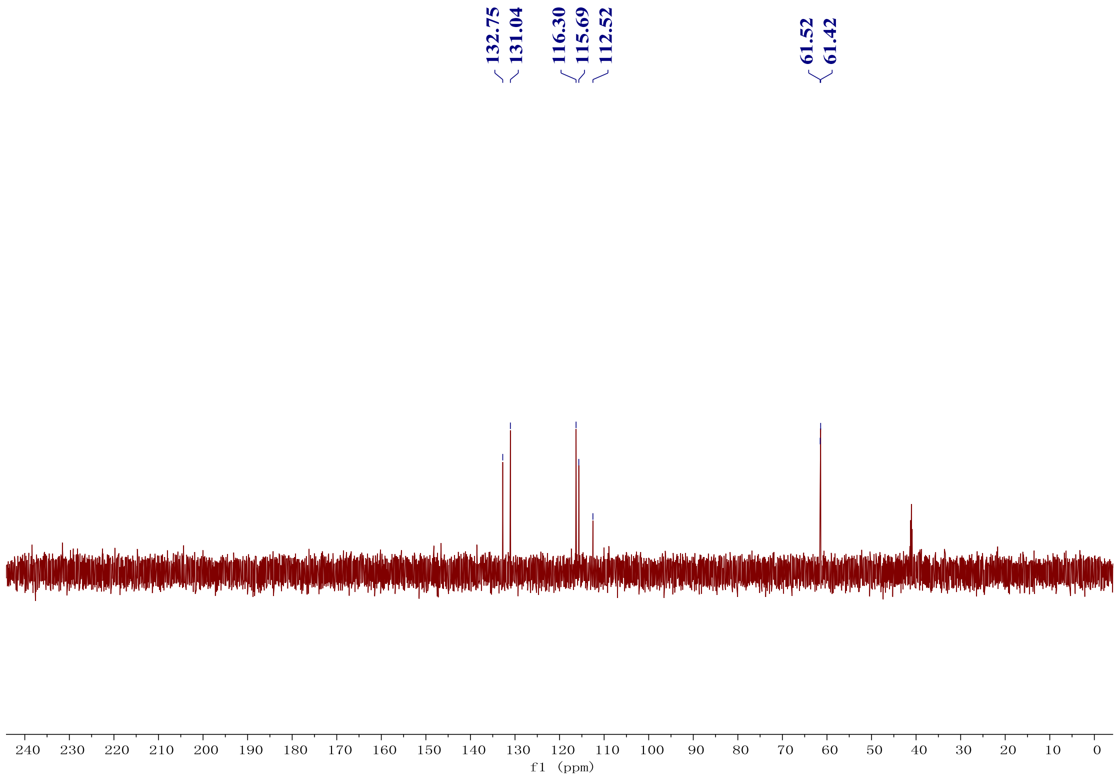

Figure S13. DEPT135 spectrum of compound **2**

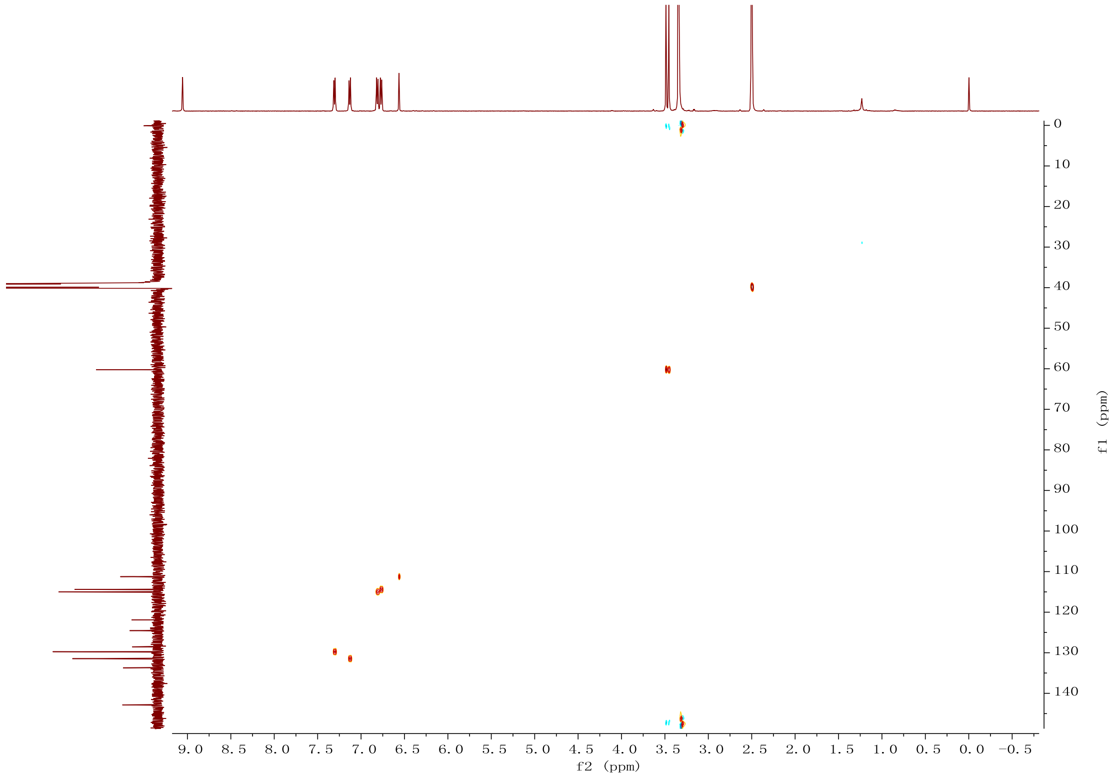

Figure S14. HSQC spectrum of compound **2**

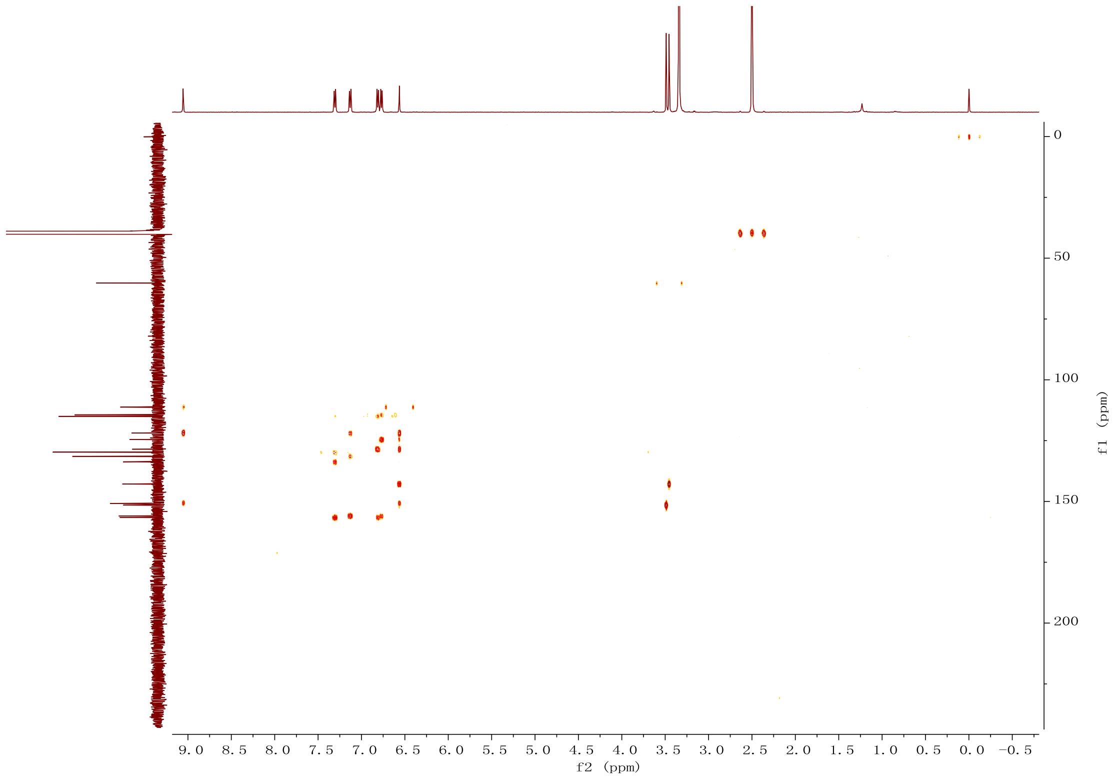

Figure S15. HMBC spectrum of compound **2**

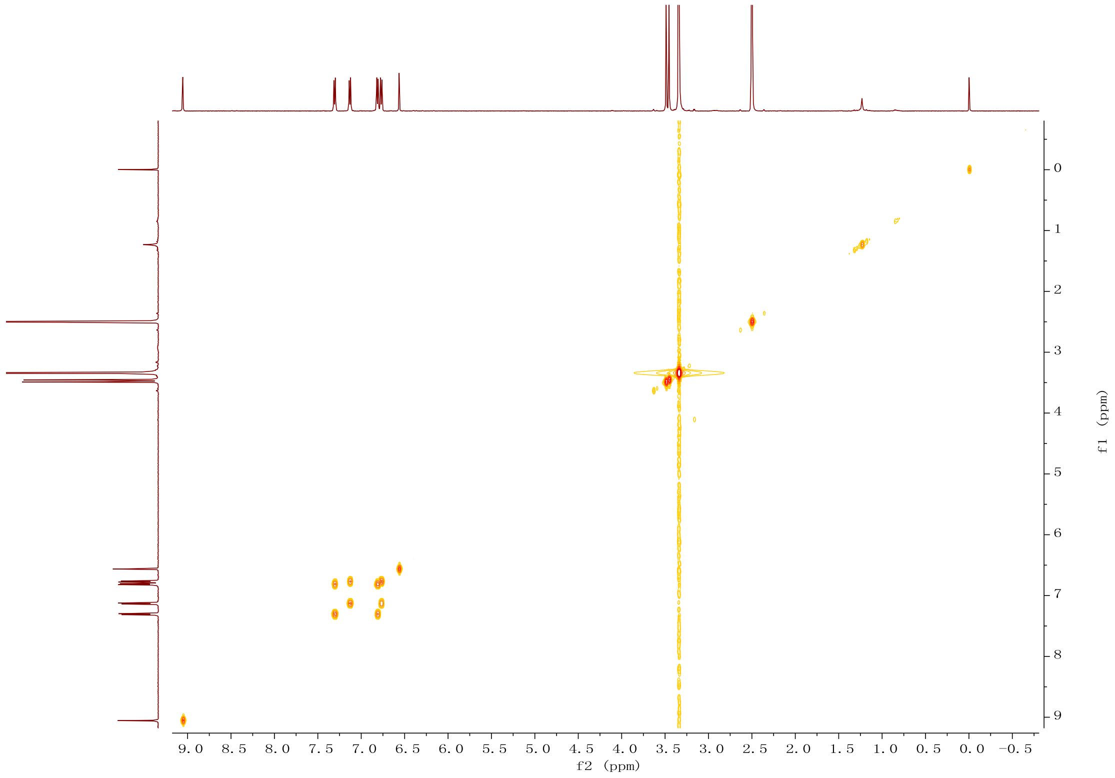

Figure S16. ^{1}H-^{1}H COSY spectrum of compound **2**

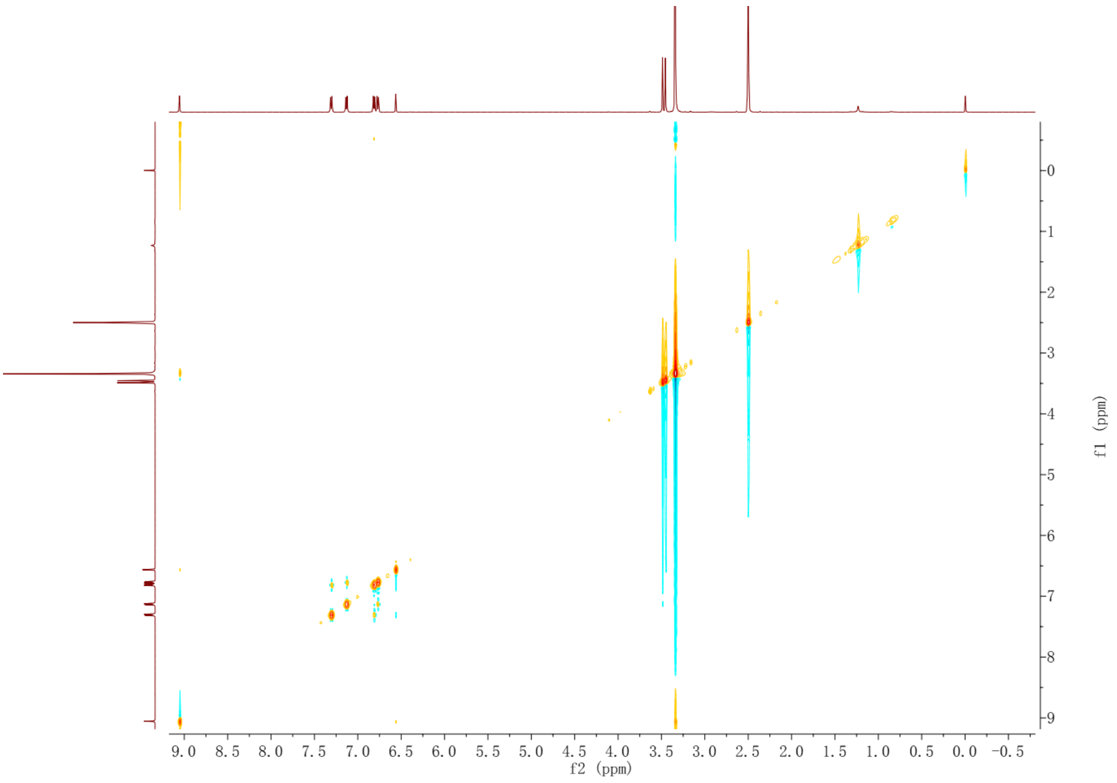

Figure S17. ROESY spectrum of compound **2**

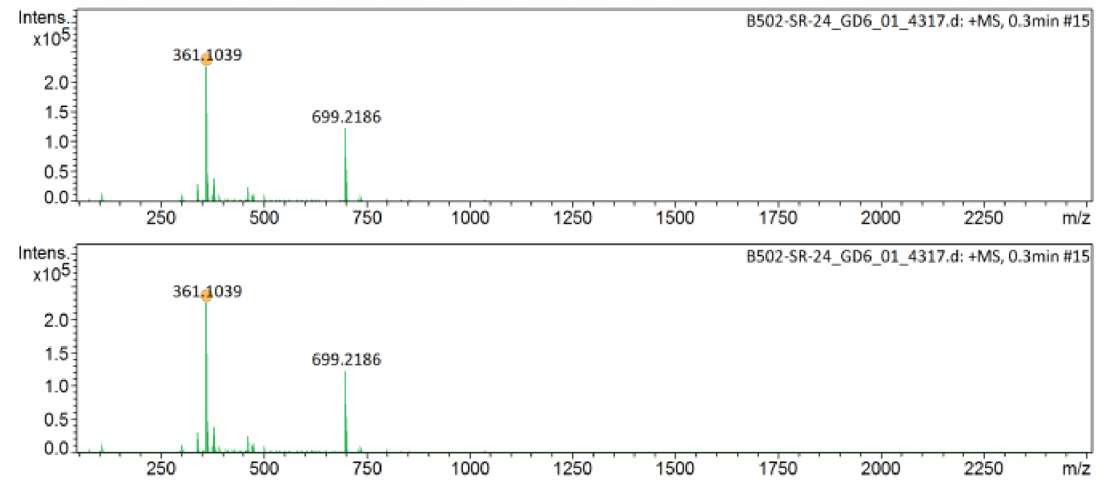

Meas. m/z	#	Ion Formula	m/z	err [ppm]	mSigma	# mSigma	Score	rdb	e⁻ Conf	N-Rule	Adduct
361.1039	1	C20H18NaO5	361.1046	2.2	7.4	1	100.00	12.0	even	ok	M+Na

Figure S18. The HRESIMS spectrum of compound **2**

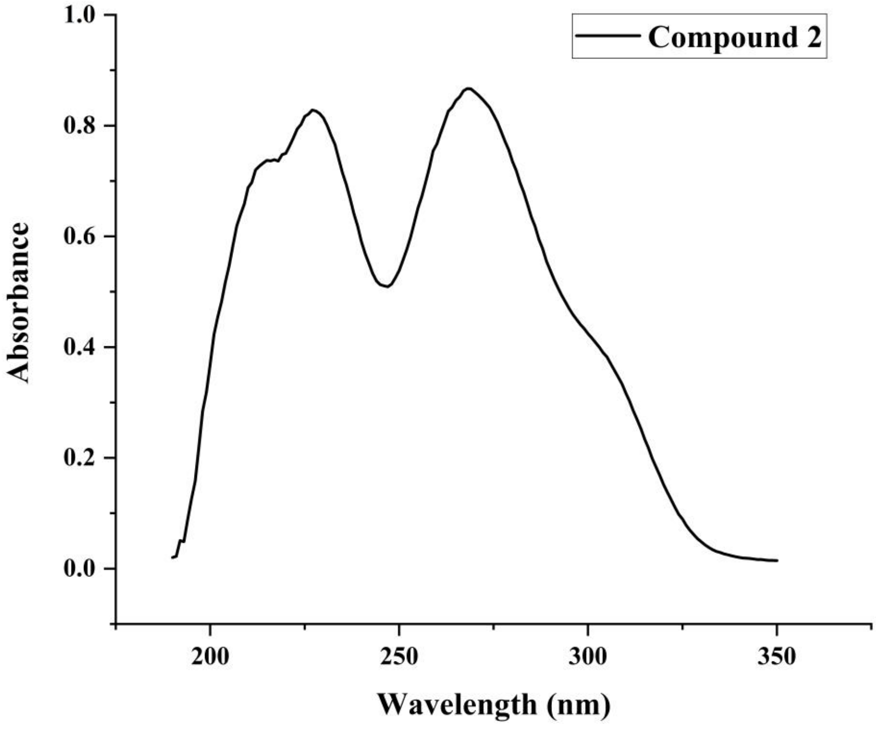

Figure S19. UV spectrum of compound **2**

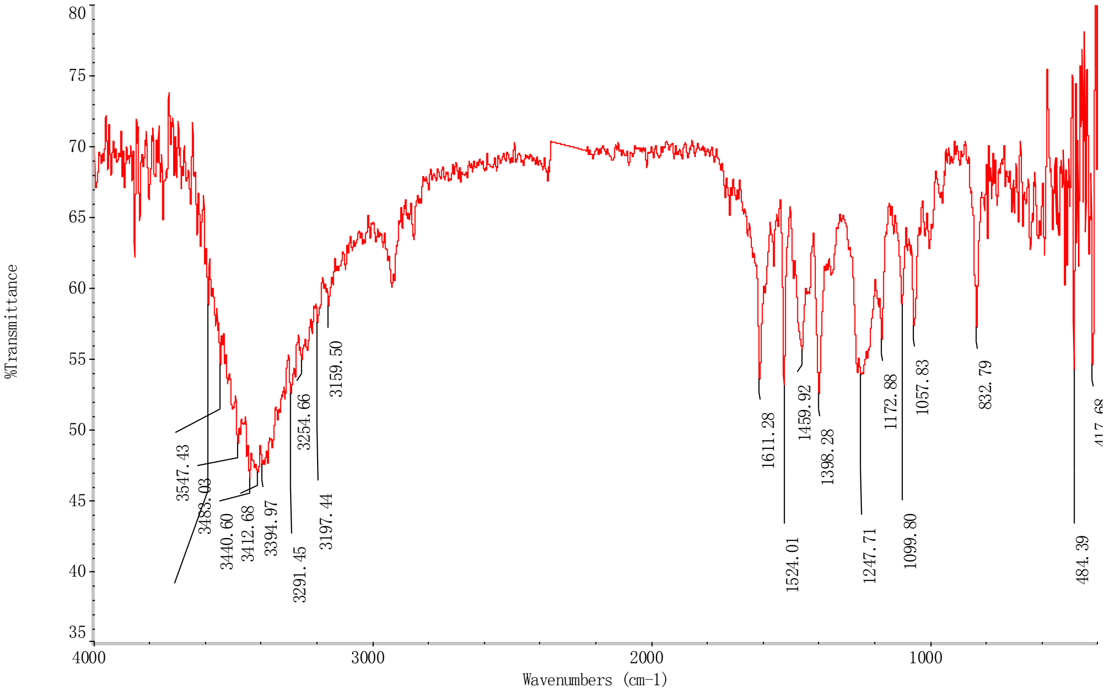

Figure S20. IR spectrum of compound **2**

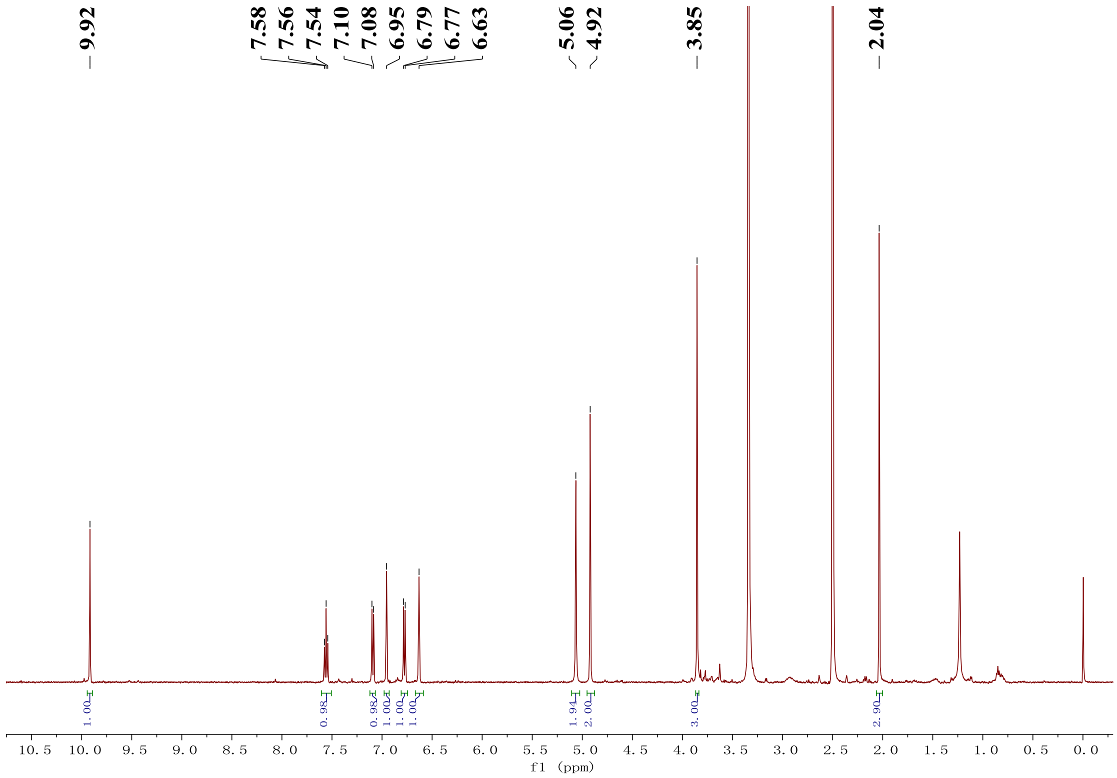

Figure S21. ^{1}H NMR (500 MHz, DMSO-d_6) spectrum of compound **3**

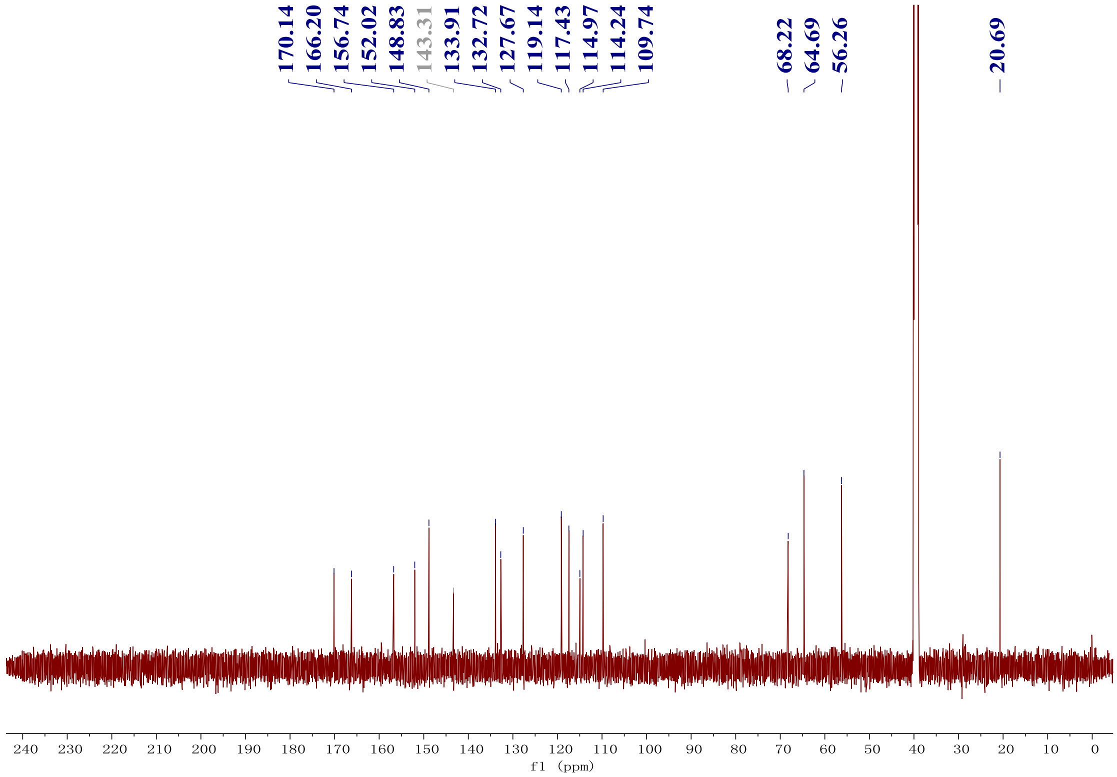

Figure S22. ^{13}C NMR (125 MHz, DMSO-d_6) spectrum of compound **3**

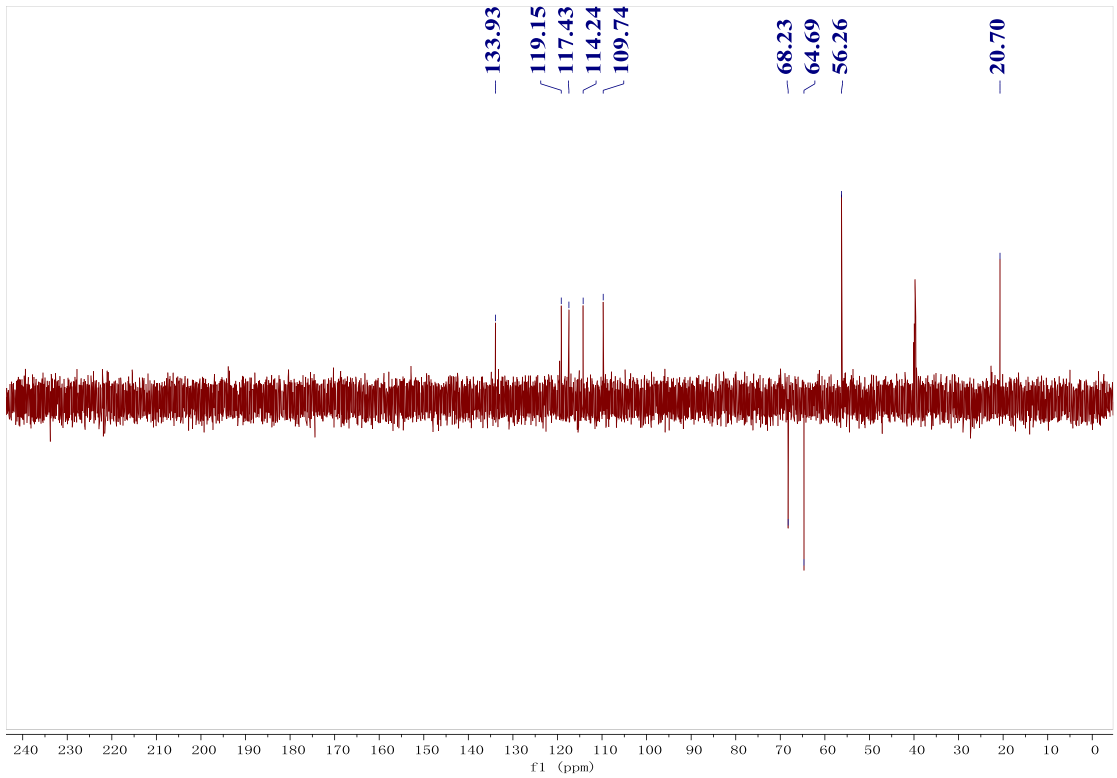

Figure S23. DEPT135 spectrum of compound **3**

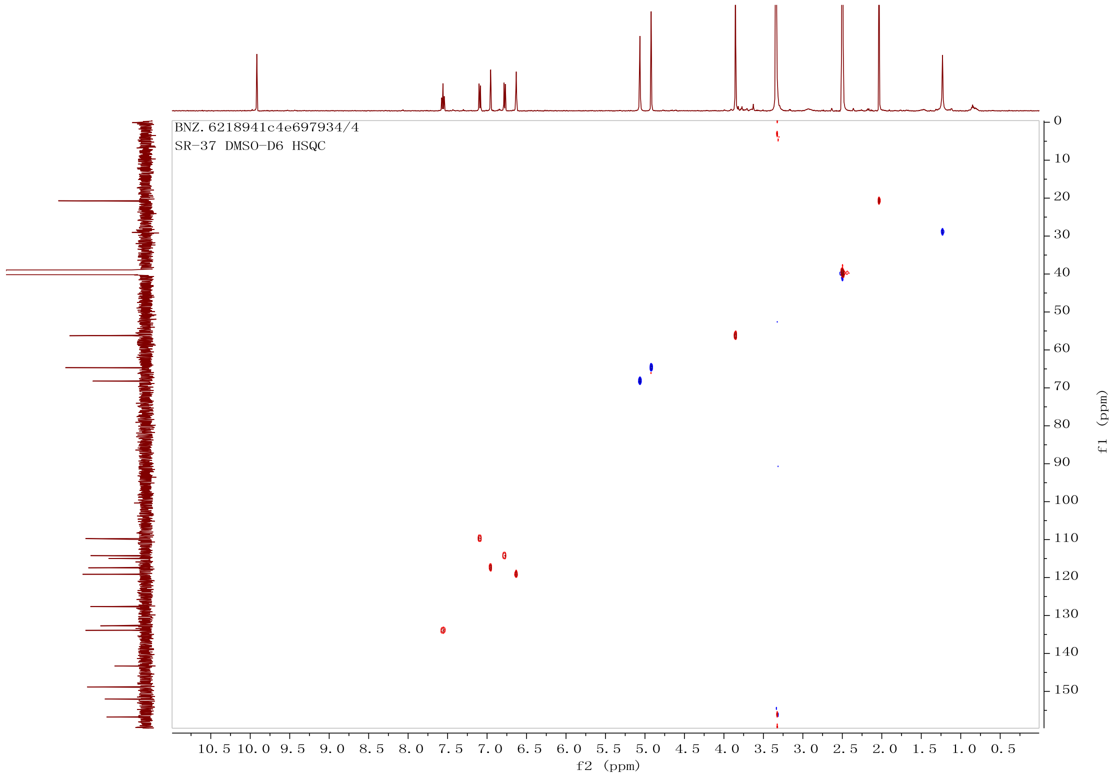

Figure S24. HSQC spectrum of compound **3**

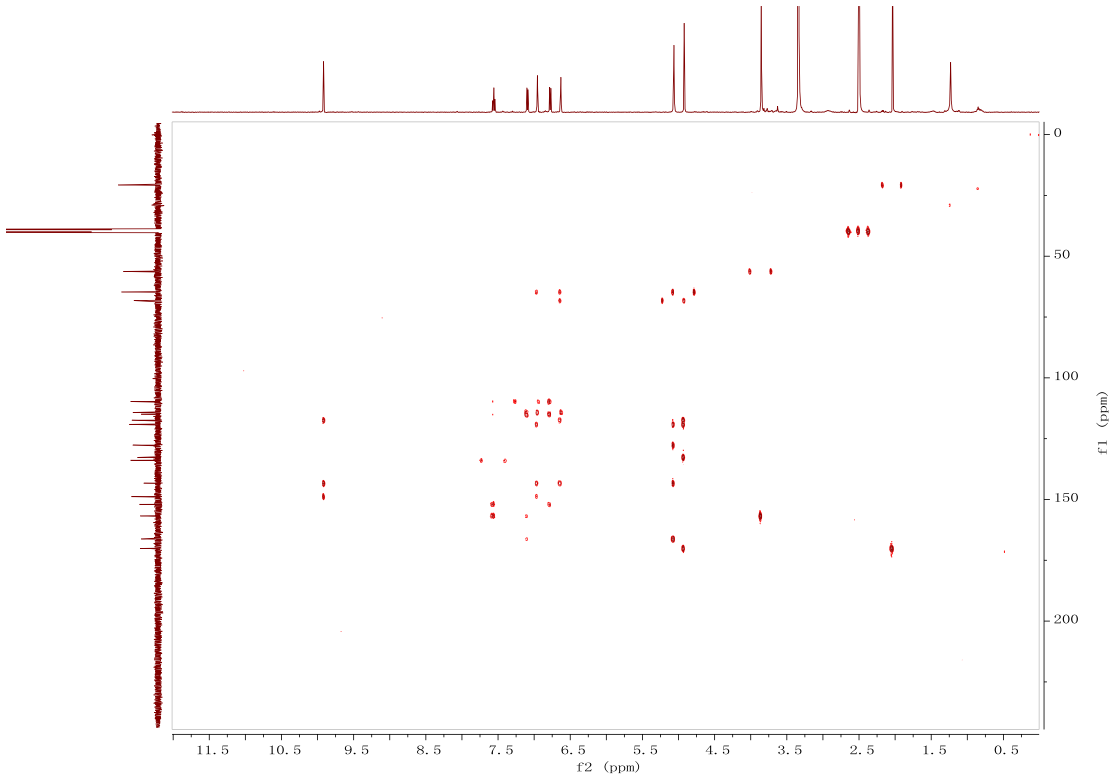

Figure S25. HMBC spectrum of compound **3**

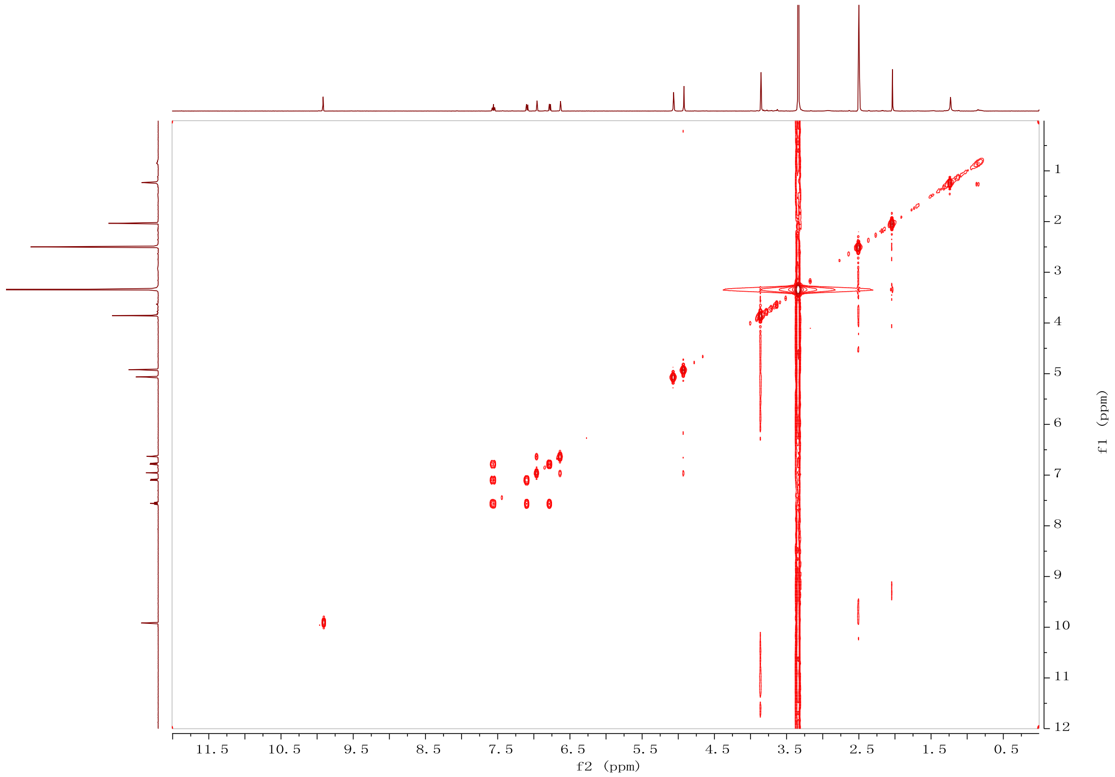

Figure S26. ^{1}H-^{1}H COSY spectrum of compound **3**

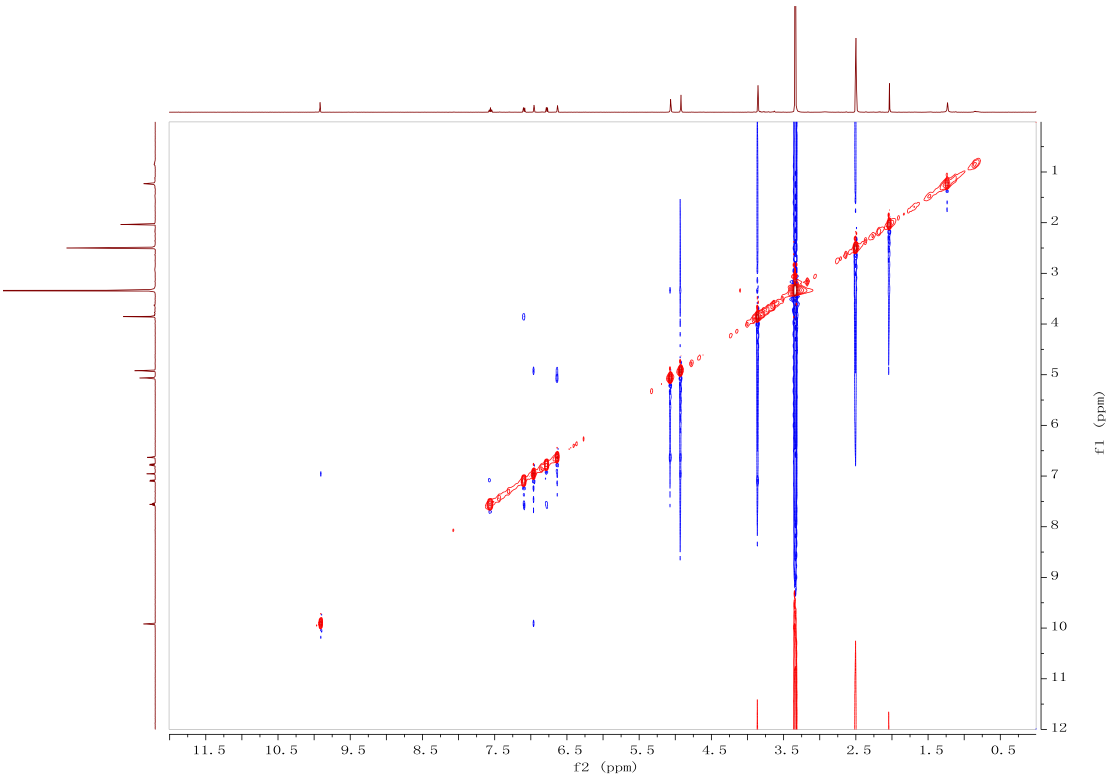

Figure S27. ROESY spectrum of compound **3**

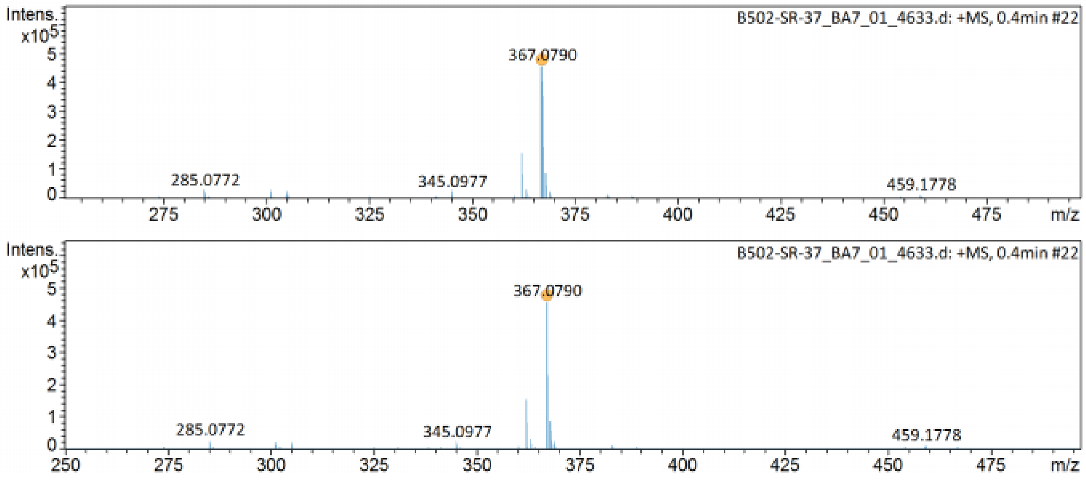

Meas. m/z	#	Ion Formula	m/z	err [ppm]	mSigma	# mSigma	Score	rdb	e^- Conf	N-Rule	Adduct
367.0790	1	C18H16NaO7	367.0788	-0.4	5.1	1	100.00	11.0	even	ok	M+Na

Figure S28. The HRESIMS spectrum of compound **3**

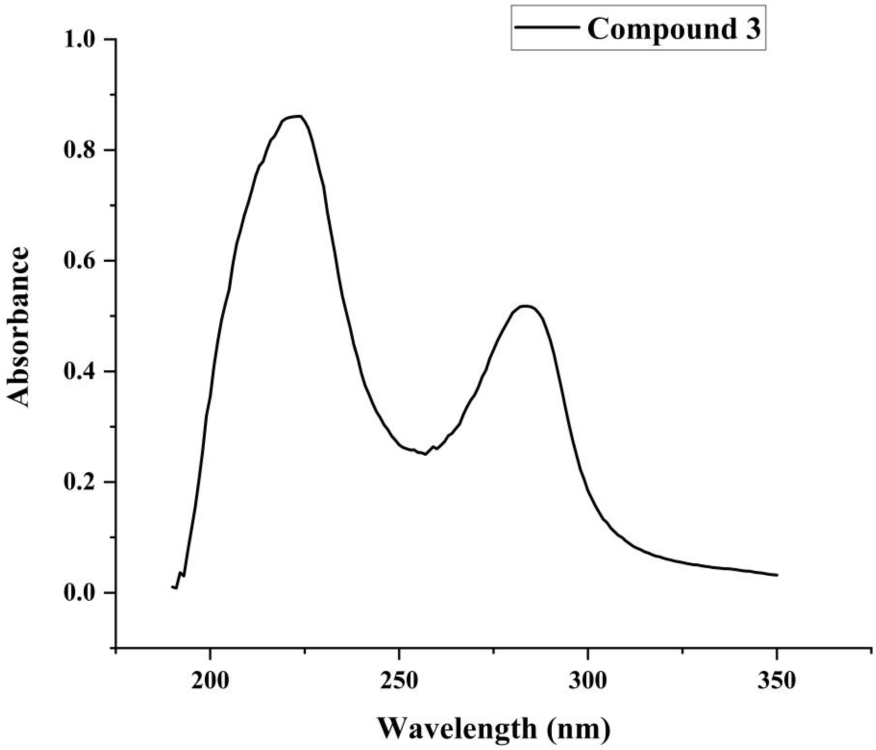

Figure S29. UV spectrum of compound **3**

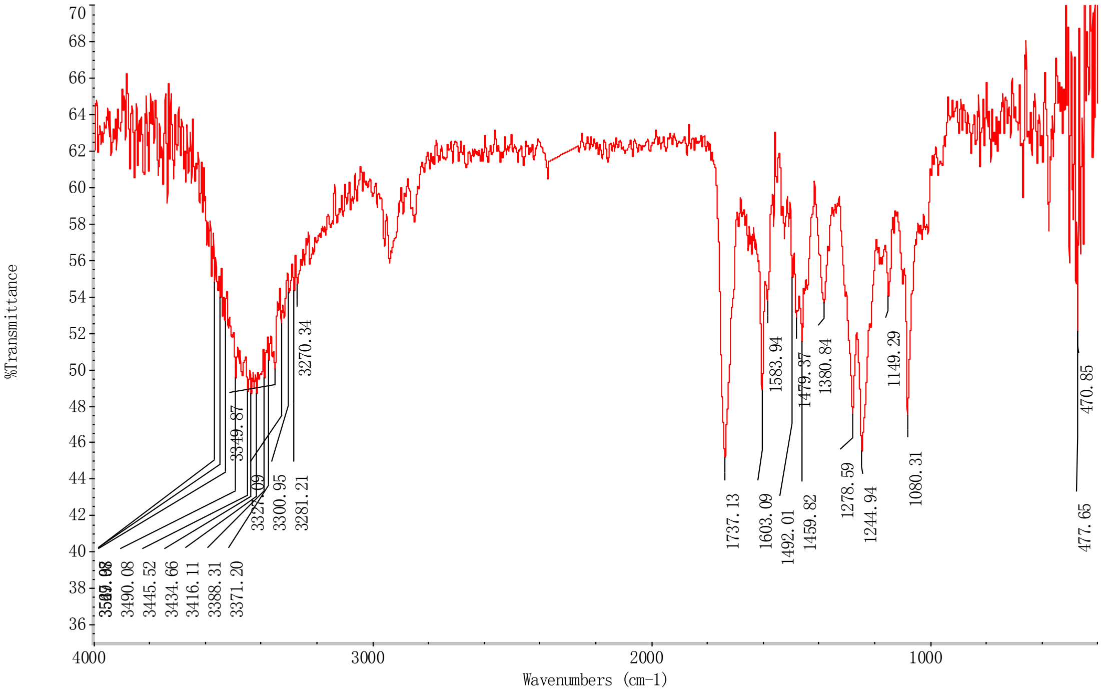

Figure S30. IR spectrum of compound **3**

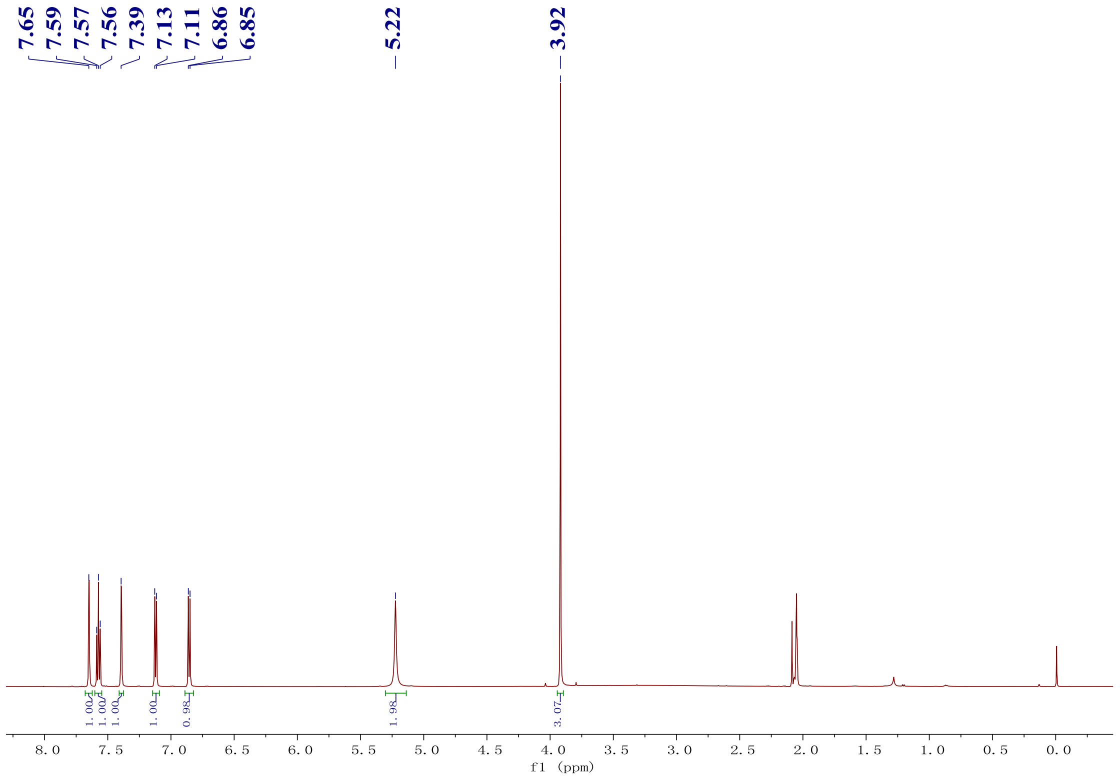

Figure S31. ^{1}H NMR (600 MHz, acetone-d_6) spectrum of compound **4**

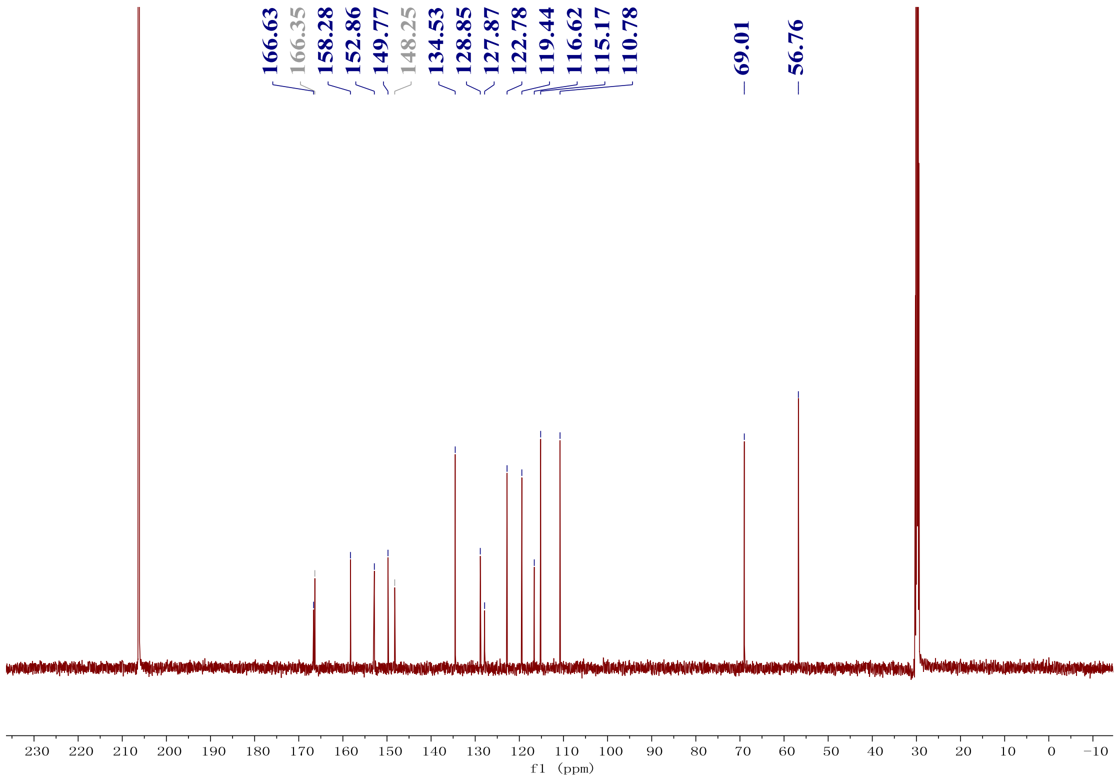

Figure S32. ^{13}C NMR (150 MHz, acetone-d_6) spectrum of compound **4**

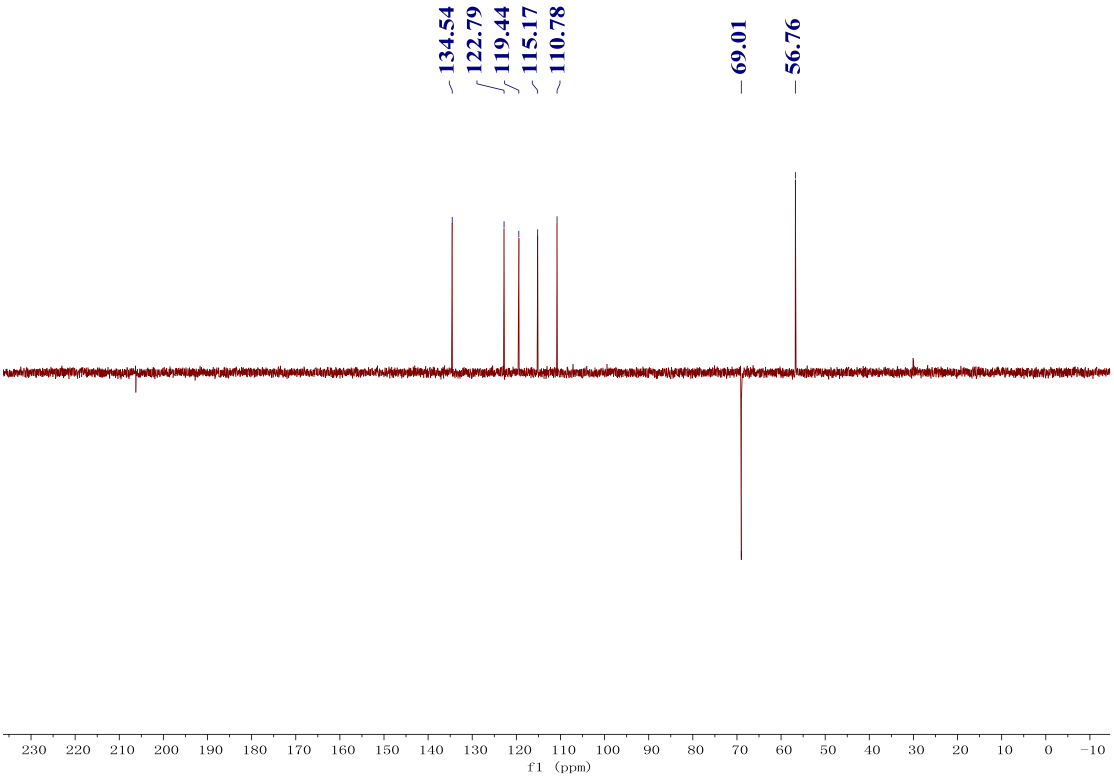

Figure S33. DEPT135 spectrum of compound **4**

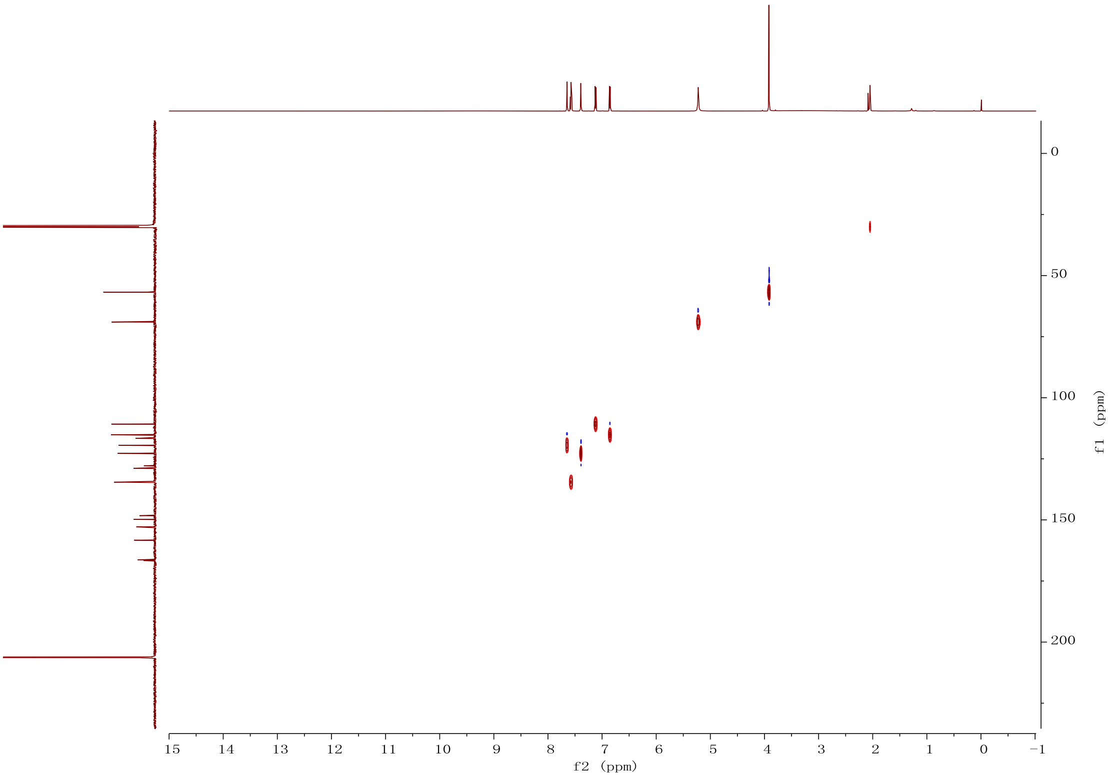

Figure S34. HSQC spectrum of compound **4**

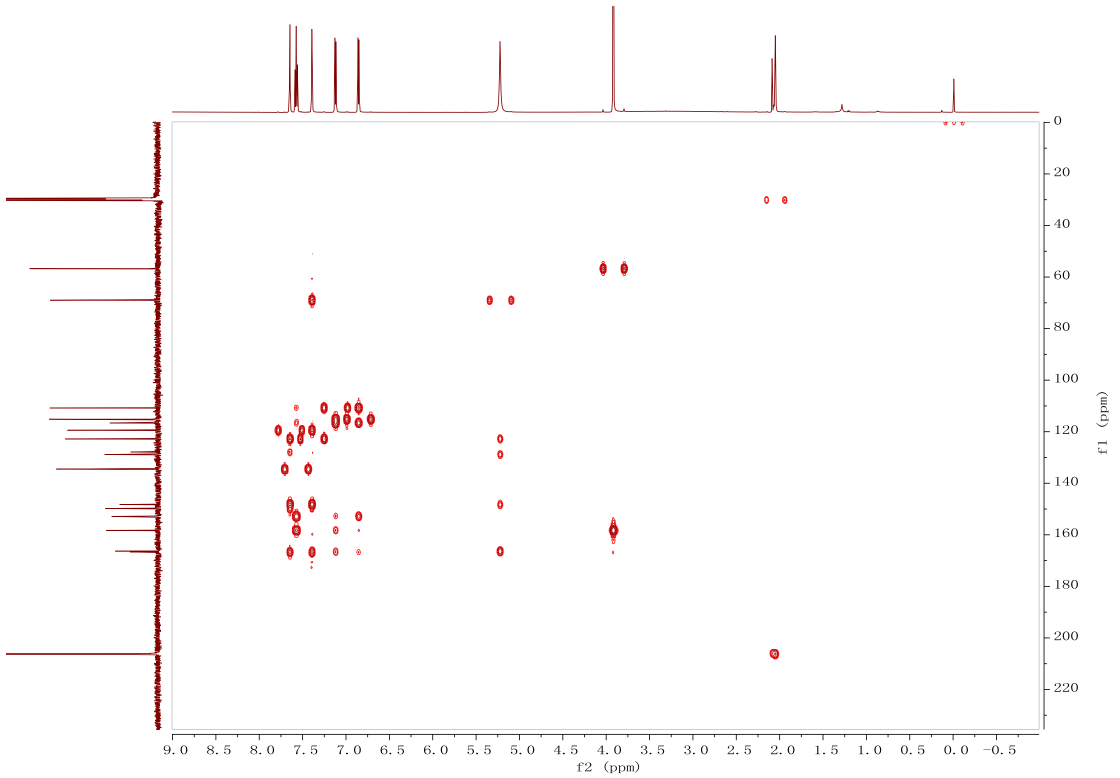

Figure S35. HMBC spectrum of compound **4**

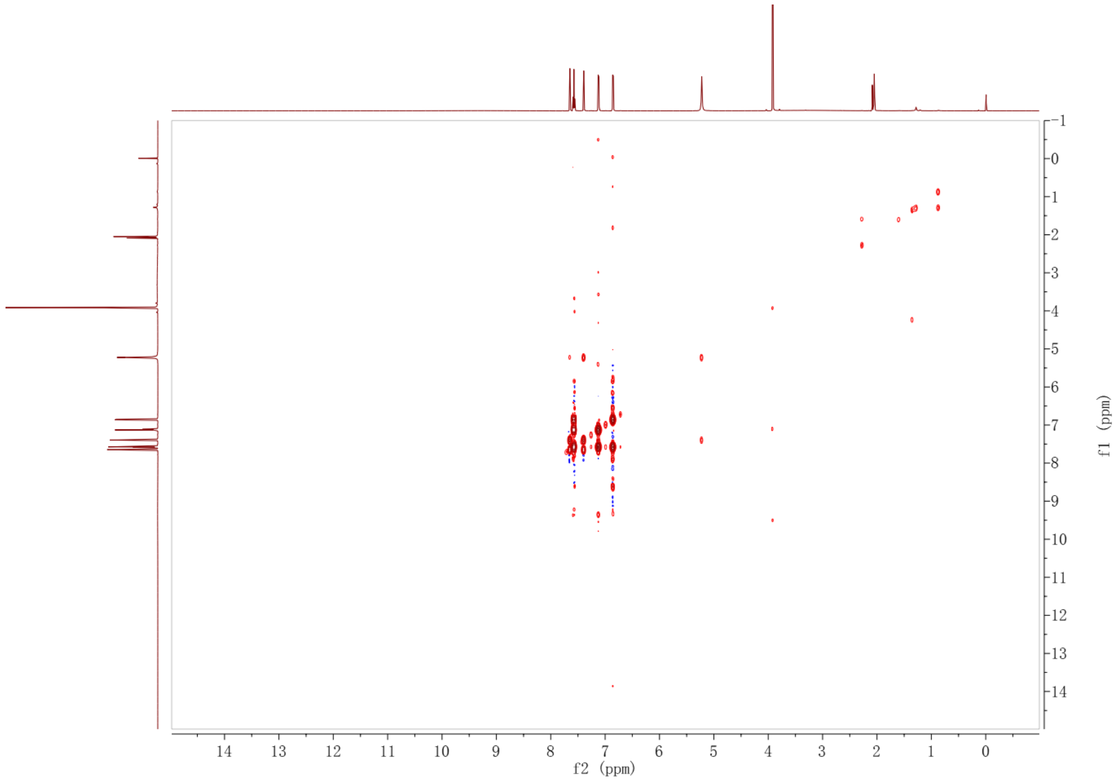

Figure S36. ^{1}H-^{1}H COSY spectrum of compound **4**

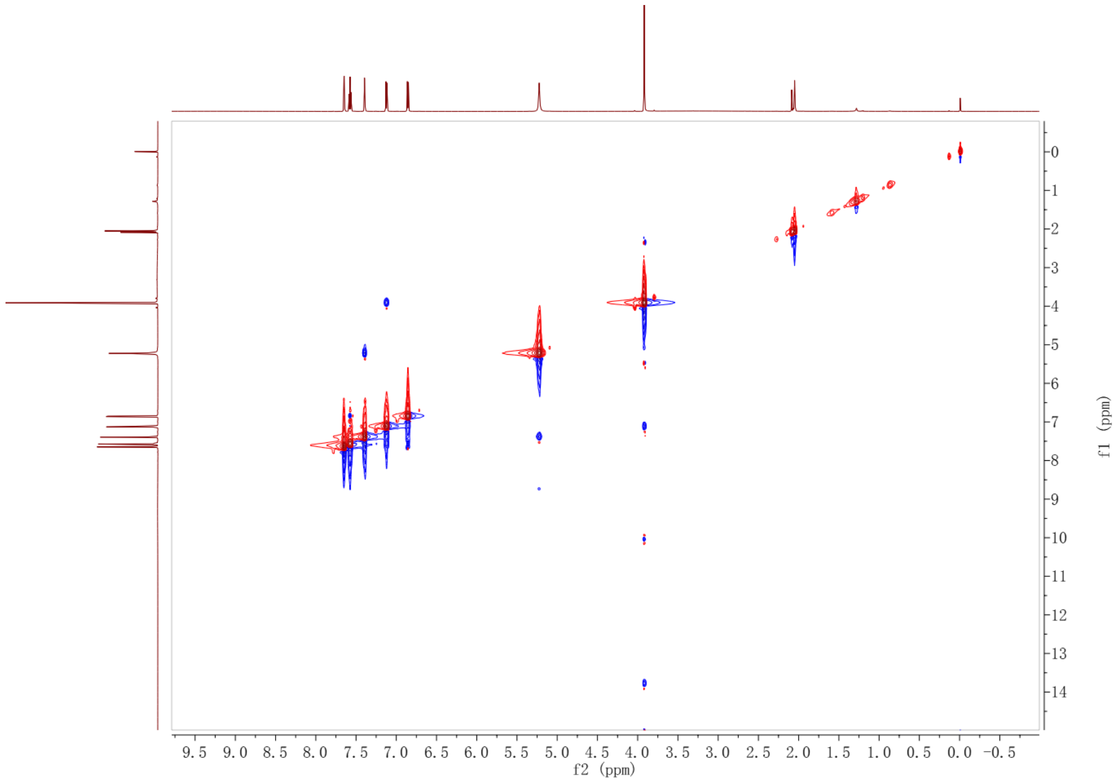

Figure S37. ROESY spectrum of compound **4**

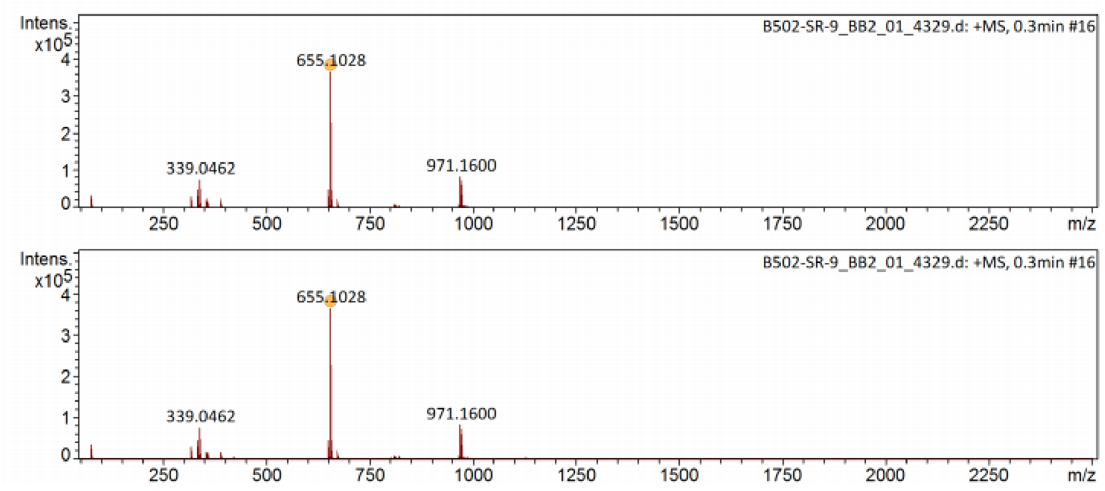

Meas. m/z	#	Ion Formula	m/z	err [ppm]	mSigma	# mSigma	Score	rdb	e⁻ Conf	N-Rule	Adduct
655.1028	1	C32H24NaO14	655.1058	4.6	2.9	1	100.00	21.0	even	ok	2M+Na

Figure S38. The HRESIMS spectrum of compound **4**

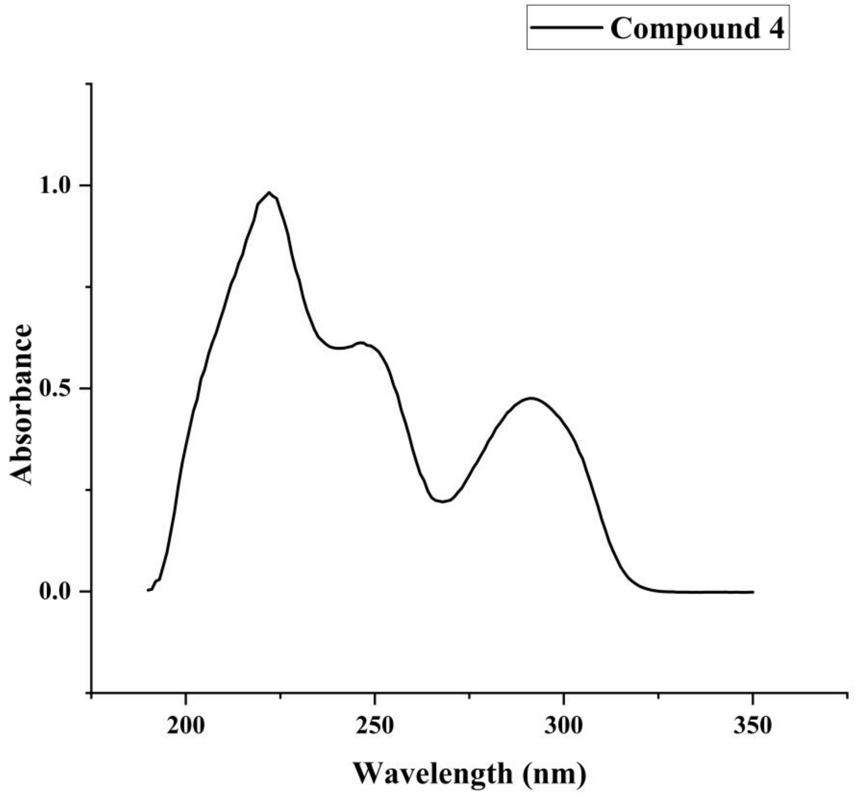

Figure S39. UV spectrum of compound **4**

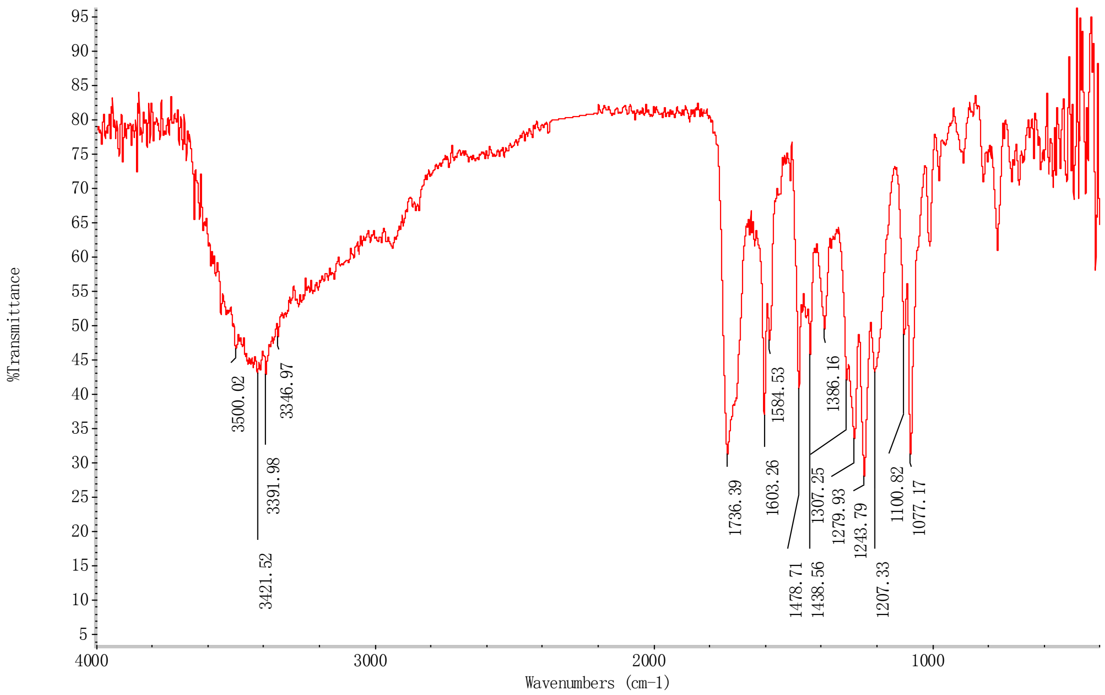

Figure S40. IR spectrum of compound **4**
